# Developments and Challenges of Miniature Piezoelectric Robots: A Review

**DOI:** 10.1002/advs.202305128

**Published:** 2023-10-27

**Authors:** Jing Li, Jie Deng, Shijing Zhang, Weishan Chen, Jie Zhao, Yingxiang Liu

**Affiliations:** ^1^ State Key Laboratory of Robotics and System Harbin Institute of Technology Harbin 150001 China

**Keywords:** miniature robot, multienvironment operation, piezoelectric actuating element, rigid and soft piezoelectric materials, working principle fusion

## Abstract

Miniature robots have been widely studied and applied in the fields of search and rescue, reconnaissance, micromanipulation, and even the interior of the human body benefiting from their highlight features of small size, light weight, and agile movement. With the development of new smart materials, many functional actuating elements have been proposed to construct miniature robots. Compared with other actuating elements, piezoelectric actuating elements have the advantages of compact structure, high power density, fast response, high resolution, and no electromagnetic interference, which make them greatly suitable for actuating miniature robots, and capture the attentions and favor of numerous scholars. In this paper, a comprehensive review of recent developments in miniature piezoelectric robots (MPRs) is provided. The MPRs are classified and summarized in detail from three aspects of operating environment, structure of piezoelectric actuating element, and working principle. In addition, new manufacturing methods and piezoelectric materials in MPRs, as well as the application situations, are sorted out and outlined. Finally, the challenges and future trends of MPRs are evaluated and discussed. It is hoped that this review will be of great assistance for determining appropriate designs and guiding future developments of MPRs, and provide a destination board to the researchers interested in MPRs.

## Graphical abstract

Miniature piezoelectric robots (MPRs) have exhibited many highlight features and captured considerable attentions and favor of numerous scholars. A comprehensive review of recent development in MPRs is provided, including the operating environment, the structure of piezoelectric actuating element, working principle, attempts of new fabricated methods and piezoelectric materials, and application progresses. The challenges and future trends are evaluated and discussed.

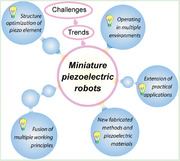

## 1 Introduction

In recent decades, miniature robots have attracted considerable attention in academic and industry due to their highlight features, including small size, light weight, low cost, and agile movement,^[^ ^1^, ^2^, ^3^, ^4^, ^5^ ^]^ compared with middle or large robots.^[^ ^6^, ^7^, ^8^ ^]^ Miniature robots here refer to the robots with a characteristic size (body length) less than 100 mm (commonly from a few millimeters to a few centimeters). These highlight features have made miniature robots be widely researched and applied in the fields of fault inspection, search and rescue, reconnaissance, micromanipulation, and even inside human body.^[^ ^9^, ^10^, ^11^, ^12^, ^13^ ^]^ Traditional miniature robots are usually driven by electromagnetic motors, which can easily achieve many advantages, such as high speed, simple control, and small volume.^[^ ^14^, ^15^, ^16^, ^17^, ^18^ ^]^ However, there are also several limitations owing to the structure and working principle of the electromagnetic motor: relatively complex structure caused by the transmission mechanism, severe torque dissipation and impeded output force due to the scaling of components, such as bearings, magnets, and coils. With the rapid development of new smart materials, many functional actuating elements for miniature robots have been presented and studied, including but not limited to pneumatic actuators,^[^ ^19^, ^20^, ^21^ ^]^ shape memory alloys (SMAs),^[^ ^22^, ^23^, ^24^ ^]^ twisted artificial muscles,^[^ ^25^, ^26^, ^27^ ^]^ dielectric elastomers,^[^ ^28^, ^29^, ^30^ ^]^ soft electrothermal actuating elements,^[^ ^31^, ^32^ ^]^ magnetostrictive actuating elements,^[^ ^33^, ^34^, ^35^ ^]^ optical actuating elements,^[^ ^36^, ^37^, ^38^ ^]^ piezoelectric actuating elements.^[^ ^39^, ^40^, ^41^, ^42^ ^]^ Among them, the piezoelectric actuating elements exhibit many impressive advantages, such as compact structure, high power density, fast response, high resolution, and no electromagnetic interference^[^ ^43^, ^44^, ^45^, ^46^, ^47^, ^48^ ^]^; these merits make the piezoelectric actuating elements greatly suitable for the development of the miniature robots, and have captured the attentions and favors of numerous scholars.^[^ ^49^, ^50^, ^51^, ^52^ ^]^

The miniature robots driven by piezoelectric ceramics, namely miniature piezoelectric robots (MPRs), can achieve movements by converting electrical energy into mechanical energy via the inverse piezoelectric effect.^[^ ^53^, ^54^, ^55^ ^]^ After decades of efforts, various MPRs with diverse mechanical structures, working principles and motion capabilities have been developed. Fortunately, MPRs have inherited the features of piezoelectric actuating elements and exhibit many unique advantages, including simple and compact structures,^[^ ^56^, ^57^, ^58^ ^]^ fast speed,^[^ ^59^, ^60^, ^61^ ^]^ high resolution,^[^ ^62^, ^63^, ^64^ ^]^ agile movements^[^ ^65^, ^66^, ^67^ ^]^ and so on. Many excellent reviews related topics of miniature robots and piezoelectric actuating elements have been separately addressed^[^ ^68^, ^69^, ^70^, ^71^, ^72^ ^]^; however, the general summaries of various MPRs have been ignored. Therefore, the aim of this review is to provide a comprehensive and timely reference for the development of various MPRs.

**Figure 1:**
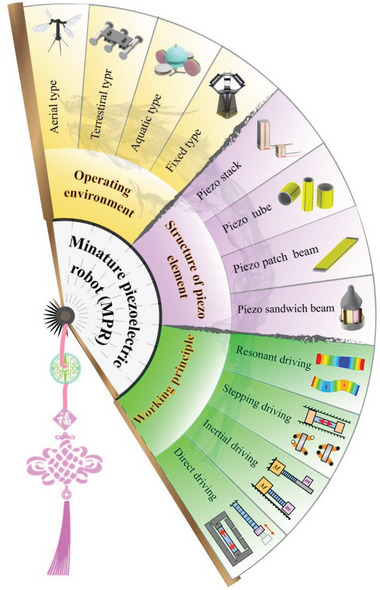
Classification of MPRs from three aspects of operating environments, the structures of piezoelectric elements, and working principles. Aerial type. Reproduced with permission.^[^ ^83^ ^]^ Copyright, 2019. Springer Nature. Terrestrial type. Reproduced with permission. ^[^ ^140^ ^]^ Copyright 2021, Wiley‐VCH. Aquatic type. Reproduced with permission.^[^ ^94^ ^]^ Copyright 2023, IEEE. Boundary fixed type. Reproduced with permission.^[^ ^106^ ^]^ Copyright 2018, AAAS.

In view of the rich variety of MPRs, we try to classify miniature piezoelectric robots from three aspects: operating environments, the structures of piezoelectric elements and working principles, as shown in **Figure** **1**. The operating environments contain air, water, ground, and boundary fixed type; and the structures of piezoelectric elements usually include the piezoelectric stack, piezoelectric tube, piezoelectric patch beam and piezoelectric sandwich beam, which are summarized in Section 2. In Section 3, we focus on the various working principles of MPRs, including resonant actuating type, direct driving type, stepping driving type, and inertial driving type. Then, the manufacturing methods and materials of MPRs and the wide applications of MPRs are given in Section 4 and Section 5, respectively. Finally, we summary the existing challenges of MPRs and discuss their future development trends in Section 6, and a conclusion is followed in Section 7.

## 2 Overview and Classifications of MPRs

MPRs are the robots driven by piezoelectric actuating elements, and their body length is generally from a few millimeters to a few centimeters. For MPRs, there are two conversion processes to achieve their continuous motions.^[^ ^73^, ^74^ ^]^ One process is to transform the applied electrical energy into micro deformations of the piezoelectric actuating elements based on the inverse piezoelectric effect; the other is to convert the micro deformations of the piezoelectric actuating elements into the micro stepping motions of MPRs by the friction, inertial, or press forces; and the continuous motions of MPRs can be achieved by accumulating the micro stepping motions. Such conversion processes based on the inverse piezoelectric effect are the unique feature of MPRs that is different from other types of miniature robots.

The inverse piezoelectric effect was discovered by the Curie brothers in 1881, and attracted much attention of many scholars. Some researchers had tried to use it to design actuators with large‐stroke motions; until the 1960s, some prototypes of piezoelectric actuators were proposed and designed to realize preliminary motions.^[^ ^75^, ^76^ ^]^ With the development of piezoelectric actuators, studies on MPRs began to appear in the 1990s.^[^ ^77^, ^78^, ^79^, ^80^ ^]^ Then, in the subsequent development of MPRs, various MPRs with different operating environments, piezoelectric actuating elements, and working principles have been proposed and designed. In this section, we will introduce and compare MPRs with different operating environments and piezoelectric actuating elements in sequence.

### 2.1 MPRs with Different Operating Environments

**Figure 2:**
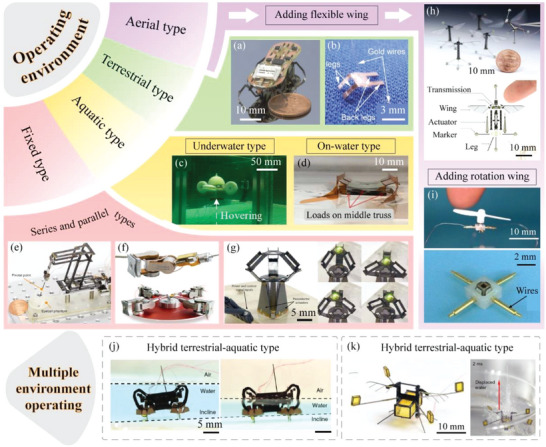
MPRs with different operating environments. a) A terrestrial‐type hexapod MPR. Reproduced with permission.^[^ ^87^ ^]^ Copyright 2011, IEEE. b) A terrestrial‐type quadrupedal MPR. Reproduced with permission.^[^ ^88^ ^]^ Copyright 2016, Springer Nature. c) An underwater‐type MPR. Reproduced with permission.^[^ ^94^ ^]^ Copyright 2023, IEEE. d) An on‐water type MPR. Reproduced with permission.^[^ ^98^ ^]^ Copyright 2021, IEEE. e) A series fixed‐type MPR. Reproduced with permission.^[^ ^104^ ^]^ Copyright 2020, Springer Nature. f) A series boundary fixed type MPR named MM3A‐EM.^[^ ^105^ ^]^ g) A parallel boundary fixed type MPR named millDelta. Reproduced with permission.^[^ ^106^ ^]^ Copyright 2018, AAAS. h) An insect‐scale, flapping‐wing MPR. Reproduced with permission.^[^ ^111^ ^]^ Copyright 2013, AAAS. i) An aerial type MPR with a rotary wing. Reproduced with permission.^[^ ^112^ ^]^ Copyright 2022, IEEE. j) A hybrid terrestrial‐aquatic MPR. Reproduced with permission.^[^ ^113^ ^]^ Copyright 2018, Springer Nature. k) A hybrid aerial‐aquatic MPR. Reproduced with permission.^[^ ^114^ ^]^ Copyright 2017, AAAS.

According to the operating environment, MPRs can be divided into the terrestrial type ones, the aquatic type ones, the boundary fixed type ones, and the aerial type ones,^[^ ^81^, ^82^, ^83^, ^84^, ^85^, ^86^ ^]^ as shown in **Figure** **2**. For the terrestrial type MPRs, most of them are designed with foot structure. The motion of the driving foot can be directly generated by the deformation of the piezoelectric actuating element, and can also be indirectly transformed through a transmission mechanism; then, the movements of MPRs can be realized by the friction force between the driving foot and the operating plane. Baisch et al.^[^ ^87^ ^]^ proposed a hexapod terrestrial MPR with length of 47 mm and wright of 1.7 g, as shown in Figure 2a; a flexure‐based spherical five‐bar hip joint was designed to transform the bending deformation of the piezoelectric bimorph actuators into the lifting and swinging motions of the driving feet, which could help the robot run on the ground with speed of 43 mm ^−1^ s. Hida et al.^[^ ^88^ ^]^ presented a quadrupedal terrestrial MPR with length of about 6.3 mm, as shown in Figure 2b; the robot could run at speed of 136 mm ^−1^ s by directly using the vibration of the piezoelectric actuating element without any transmission mechanism. The aquatic type MPRs can be further divided into the underwater type ones and the on‐water type ones. For the underwater type MPRs, there are usually two methods to generate the underwater actuating force. One method is to utilize the flexible piezoelectric actuating element to imitate the swing of the fish tail,^[^ ^89^, ^90^ ^]^ the other method is to combine the piezoelectric actuating element with a microporous structure or propeller to generate the required propulsion.^[^ ^91^, ^92^ ^]^ For example, Junqiang et al.^[^ ^93^ ^]^ proposed a bionic robotic fish with resonant actuation of a soft piezoelectric actuating element, the generated micro thrust was measured, and the pressure field evolution was simulated by CFD (computational fluid dynamics). Zhou et al.^[^ ^94^ ^]^ designed a cross‐shaped underwater MPR with four piezoelectric pulse‐jet actuators, as shown in Figure 2c, the robot achieved floating, sinking, and hovering motions in the vertical direction, and linear, rotary, and turning motions in the horizontal direction. The on‐water type MPRs usually use their own buoyancy or surface tension to float on the water surface, and there are also usually two approaches to obtain the actuating force on the water surface. One approach is to design paddle‐like structures to push the water, and the other approach is to add flap mechanisms that flaps the air to drive the robot.^[^ ^95^, ^96^, ^97^ ^]^ For instance, Du et al.^[^ ^98^ ^]^ presented an on‐water type MPR with length of 40 mm, as shown in Figure 2d, the robot was designed with four hydrofoils to paddle the water, and could swim with a maximum speed of 20 mm ^−1^ s. Zhou et al.^[^ ^99^ ^]^ proposed an insect‐inspired on‐water type MPR, which was designed with two flapping wings to generate the actuating force, and could skate on the water surface with a maximum speed of 151 mm ^−1^ s. For the boundary fixed type MPRs, one end of the robot is fixed, and the other end can realize multi‐DOF motions. According to the layout of the piezoelectric drive elements, the boundary fixed type MPRs can usually be divided into the series ones and the parallel ones.^[^ ^100^, ^101^, ^102^, ^103^ ^]^ The series boundary fixed type MPRs have the advantages of large working range, simple structure, and easy control. Suzuki and Wood^[^ ^104^ ^]^ proposed a series boundary fixed type MPR inspired by origami, as shown in Figure 2e, the robot had a size of 50 × 70 × 50 mm^3^ and a weight of 2.4 g, and could operate with a working range of 0.5 mm^2^, a positional precision of 26.4 µm, and a payload capacity of 27 mN. Besides, Kleindiek Nanotechnik company (Germany) designed a series boundary fixed type MPR named MM3A‐EM,^[^ ^105^ ^]^ as shown in Figure 2f; the robot can output two‐DOF rotary motions with a large range of 360°, an angel resolution of 0.1°, and torque of 0.01 mN m. On the other hand, the parallel boundary fixed type MPRs have the advantages of high stiffness, large load capacity, high precision, and small inertial force of the end part. As shown in Figure 2g, McClintock et al.^[^ ^106^ ^]^ presented a parallel boundary fixed type MPR named millDelta, the robot was limited in a size of 15 × 15 × 15 mm^3^ and a weight of 0.43 g; the achieved workspace, position precision, and maximum load were 7.01 mm^3^, 5 µm and 1.31 g, respectively. For the aerial type MPRs, there are usually two methods to realize the required lift force. One method is to add the flexible wings to flap the air, and the other method is to combine the piezoelectric actuating element with a rotation‐wing mechanism to achieve the lift force.^[^ ^107^, ^108^, ^109^, ^110^ ^]^ For instance, Wood et al.^[^ ^111^ ^]^ proposed an insect‐scale, flapping‐wing MPR with only 0.08 g, as shown in Figure 2h; the robot was designed with two passive rotation wing hinges to transform the bending vibration of the piezoelectric actuating element to the flapping motion of the wing. Chiang et al.^[^ ^112^ ^]^ presented an aerial‐type MPR with a rotary wing, as shown in Figure 2i; the robot used a rotary piezoelectric actuator to drive the rotary wing, and the angular speed was over 1800 rad s^‐1^.

As mentioned above, MPRs could exhibit good performance in the different operating environment by combing the corresponding auxiliary structures, such as the foot, the microporous jet, the flexible wing, and so on. Moreover, some researchers have studied MPRs operating in multiple environments to further improve the environmental adaptability. As shown in Figure 2j, Chen et al.^[^ ^113^ ^]^ presented a hybrid terrestrial‐aquatic MPR; the robot could be supported on the water surface by the combination of surface tension and buoyancy of four electrowetting pad (EWP), and could swim forward and turn by using the passive flaps; the robot could transition from water surface to water bottom by using the EWP to break the water surface, and then move to the land by climbing a modest incline. Moreover, Wood et al.^[^ ^114^ ^]^ proposed a hybrid aerial‐aquatic MPR with weight of 0.175 g, as shown in Figure 2k; a lightweight device that integrated electrolytic plates and a sparker was designed to overcome the surface tension, helping the robot transition from water to air. Although there have been related studies, there are still few reports on MPRs that operate in multiple environments. How to better realize the movement and transition of MPRs in multiple environments is an interesting research topic, which can help the robots improve the environment adaptability and expand their application scope. In addition, the research on the terrestrial type MPRs accounts for the vast majority, thus, the following introduction focuses on the terrestrial type MPRs.

### 2.2 MPRs with Different Piezoelectric Actuating Elements

**Figure 3:**
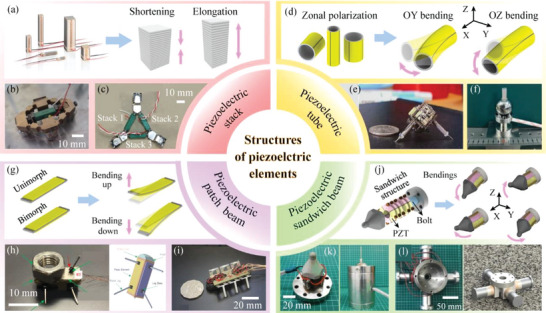
MPRs driven by different piezoelectric actuating elements. a) The common structure and deformation principle of piezoelectric stack. b) A quadrupedal MPR driven by one piezoelectric stack. Reproduced with permission.^[^ ^121^ ^]^ Copyright 2005, IOP Publishing. c) A tripodal MPR driven by three piezoelectric stacks. Reproduced with permission.^[^ ^122^ ^]^ Copyright 2018, Fuji Technology Press. d) The common structure and deformation principle of piezoelectric tube. e) A quadrupedal MPR driven by one piezoelectric stack. Reproduced with permission.^[^ ^125^ ^]^ Copyright 2001, Society of Photo‐Optical Instrumentation Engineers. f) A tripodal MPR driven by three piezoelectric stacks. Reproduced with permission.^[^ ^128^ ^]^ Copyright 2022, IEEE. g) The common structure and deformation principle of piezoelectric patch beam. h) An MPR driven by three piezoelectric patch beams. Reproduced with permission.^[^ ^131^ ^]^ Copyright 2022, Elsevier. i) A hexapod MPR driven by twelve piezoelectric patch beams. Reproduced with permission.^[^ ^132^ ^]^ Copyright 2016, IEEE. j) The common structure and deformation principle of piezoelectric sandwich beam. k) A fixed‐type MPR driven by one piezoelectric sandwich beam. Reproduced with permission.^[^ ^135^ ^]^ Copyright 2018, IEEE. l) A quadrupedal MPR driven by four piezoelectric sandwich beams. Reproduced with permission.^[^ ^138^ ^]^ Copyright 2019, IEEE.

The piezoelectric actuating element, used to achieve the conversion between the electrical and mechanical energy, is the core component of MPRs. With the development of MPRs, many piezoelectric actuating elements with different structures have been designed and applied in MPRs. As illustrated in **Figure** **3**, there are four kinds of common piezoelectric actuating elements, including piezoelectric stack, piezoelectric tube, piezoelectric patch beam, and piezoelectric sandwich beam.^[^ ^115^, ^116^, ^117^, ^118^ ^]^ Among them, the piezoelectric stack and piezoelectric tube have been designed into mature commercial products with a series of dimensional parameters; while the piezoelectric patch and sandwich beams are usually fabricated in small batches according to the specific requirements of MPRs. Figure 3a shows the common commercial piezoelectric stacks, which are formed by stacking multiple layers of piezoelectric ceramic sheets and can output their superposition deformation along the superposition direction.^[^ ^119^, ^120^ ^]^ For MPRs driven by piezoelectric stacks, the piezoelectric stacks are usually horizontally arranged inside the MPR bodies to make their driving feet generate horizontal displacements; while the vertical displacements utilized to lift and lower their driving feet are generally replaced by the on‐off of electromagnet on each foot. Yan et al.^[^ ^121^ ^]^ proposed a quadrupedal MPR driven by only one piezoelectric stack, as shown in Figure 3b, a rhombic flexure hinge mechanism and four electromagnetic legs were utilized to achieve 3‐DOF plane movements with high resolution. Torii et al.^[^ ^122^ ^]^ presented a tripodal MPR driven by three piezoelectric stacks, as illustrated in Figure 3c; similarly, its three legs were separately designed with electromagnetic elements, and the 3‐DOF plane motions could be realized by the coordination of the piezoelectric stacks and the electromagnetic feet. Figure 3d illustrates the common structure and deformation principle of the piezoelectric tube. Unlike the piezoelectric stack made of multiple ceramic sheets, the piezoelectric tube is an integral thin cylinder tube polarized from its radial direction. The inner surface of the piezoelectric tube is a continuous electrode, while the outer surface is divided into multiple partition electrodes to achieve bending deformations in multiple directions.^[^ ^123^, ^124^ ^]^ For MPRs driven by piezoelectric tubes, their piezoelectric tubes are set with four partition electrodes to realize bending deformation in two orthogonal directions and are usually used as leg structures to produce the lifting and swinging displacements of the driving feet. As shown in Figure 3e, Martel et al.^[^ ^125^, ^126^, ^127^ ^]^ presented a tripodal MPR, named Nano Walker, in which three piezoelectric tubes were used as the legs and were arranged in the circumferential direction. Gao et al.^[^ ^128^ ^]^ proposed a boundary fixed type MPR illustrated in Figure 3f, which could realize 2‐DOF rotary‐rotary motion in two orthogonal directions by utilizing the bending deformations of single piezoelectric tube. Then, the common structure and deformation principle of piezoelectric patch beam are shown in Figure 3g. The piezoelectric patch beams can be easily formed by attaching the piezoelectric ceramic sheets on a metal substrate, and can be usually divided into unimorph and bimorph beams according to whether the piezoelectric ceramic sheets are attached to both sides of the metal substrate; besides, the piezoelectric patch beams adopt the d_31_ mode of piezoelectric ceramic sheets, and can generate bending deformations in one direction.^[^ ^129^, ^130^ ^]^ For MPRs driven by piezoelectric patch beams, their piezoelectric patch beams can be used not only as bodies of MPRs but also legs, and their structural design are generally simple and flexible. As illustrated in Figure 3h, Wang et al.^[^ ^131^ ^]^ proposed T‐phage inspired MPR with a triangular prism body, which was composed of three unimorph beams and could transfer the vibrations of the unimorph beams to its driving feet and further to realize the linear and steering motions. Rios et al.^[^ ^132^ ^]^ designed a hexapod MPR named MinRAR V1, as shown in Figure 3i, in which each leg consisted of two bimorph beams to achieve the lifting and swinging motions, respectively. Then, Figure 3j shows the common structure and deformation principle of piezoelectric sandwich beam, which is generally a sandwich structure with two metal caps holding the piezoelectric ceramic sheets in the middle. Similar to the piezoelectric tubes in MPRs, the piezoelectric sandwich beams are also used as the legs of MPRs; besides, their piezoelectric ceramics work with their d_33_ mode and are set with four partition electrodes to achieve bending deformations of the leg in two orthogonal directions.^[^ ^133^, ^134^ ^]^ Zhang et al.^[^ ^135^ ^]^ presented a fixed‐type MPR shown in Figure 3k, which was actuated by only one piezoelectric sandwich beam and could realize 2‐DOF rotary‐rotary motions. Deng et al.^[^ ^136^, ^137^, ^138^ ^]^ proposed a quadrupedal MPR with four piezoelectric sandwich beams, as illustrated in Figure 3l; in which each piezoelectric sandwich beams was used as a leg to achieve lifting and swinging motions of the driving foot.

**Figure 4:**
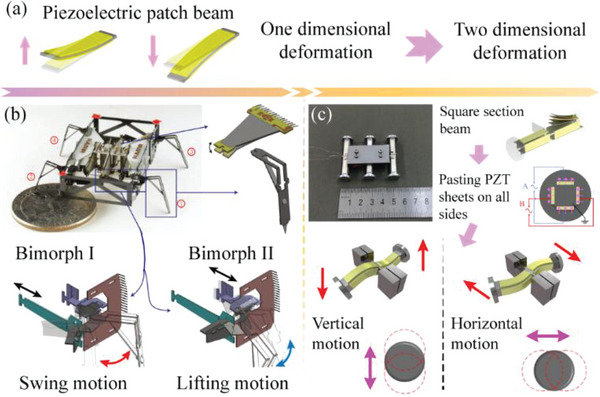
Methods for upgrading the 1‐DOF motion of the driving foot to 2‐DOF motions. a) The common structures and deformations of piezoelectric patch beam. b) Using two piezoelectric patch beams to obtain the lifting and swinging motions of a driving foot. Reproduced with permission.^[^ ^139^ ^]^ Copyright 2013, IEEE. c) A novel piezoelectric patch beam with a square section to generate 2‐DOF bending deformations. Reproduced with permission.^[^ ^140^ ^]^ Copyright 2021, Wiley‐VCH.

**Table** **1** lists and summarizes the characteristics of MPRs with different piezoelectric actuating elements. Generally, the piezoelectric patch beam has the simplest structure, which is conducive to the miniature design of the piezoelectric robot; however, the piezoelectric patch beam only realizes 1‐DOF bending deformation, which can usually limit motion agility of the driving foot. Therefore, some scholars have studied the methods that improve the 1‐DOF motion of the driving foot actuated by piezoelectric patch beam to 2‐DOF motions shown in **Figure** **4**a. One method is to increase the number of piezoelectric patch beams. Ozcan et al.^[^ ^139^ ^]^ utilized two piezoelectric patch beams to obtain the lifting and swinging motions of the driving foot, respectively, as shown in Figure 4b. Another method is illustrated in Figure 4c, Liu et al.^[^ ^140^ ^]^ proposed a novel piezoelectric patch beam with a square section, which was attached with piezoelectric ceramic sheets on all four sides to generate 2‐DOF bending deformation, thus, it could be used as a leg to achieve lifting and swinging motions of the driving feet.

**Table 1:** Advantages and disadvantages of different piezoelectric actuating element in MPRs (Note: The characteristics here are a qualitative summary of the common characteristics of this type MPR, and excluding some specially designed examples).

Piezoelectric actuating elements	Advantages	Disadvantages
Piezoelectric stack	Large deformation, relatively large output force, and being mature commercial product	Low frequency bandwidth, strict assembly and signal requirements, unidirectional deformation, and expensive cost
Piezoelectric tube	Compact structure, and being mature commercial product	Relatively complex assembly, relatively small deformation, and relatively expensive cost
Piezoelectric patch beam	Simple and compact structure, large deformation, and easy fabrication	Usually low stiffness, low frequency bandwidth, and only unidirectional deformation
Piezoelectric sandwich beam	Large output force and structure stiffness, high frequency bandwidth, loose signal requirements	Relatively small deformation, complex and large structure, and complex assembly process

## 3 MPRs with Different Working Principles

**Figure 5:**
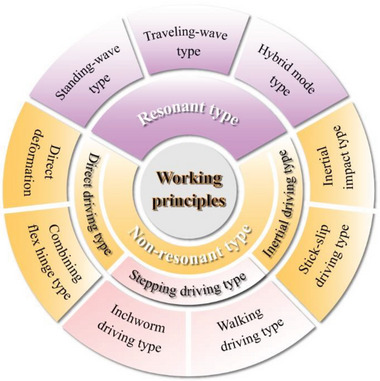
Classification of MPRs according to the used working principles, mainly including the resonant type, the direct driving type, the stepping driving type, and the inertial driving type.

With the development of MPRs, various working principles have been applied to MPRs. As plotted in **Figure** **5**, MPRs can be divided into the resonant type ones and nonresonant type ones in the light of whether the piezoelectric actuating elements work at their resonant mode, and the non‐resonant type MPRs can be further divided into direct driving type ones, stepping driving type ones and inertial actuating ones.^[^ ^141^, ^142^, ^143^, ^144^, ^145^, ^146^ ^]^ In this section, we will systematically sort out and introduce MPRs with different working principles and compare their characteristics.

### 3.1 The Resonant Type MPRs

**Figure 6:**
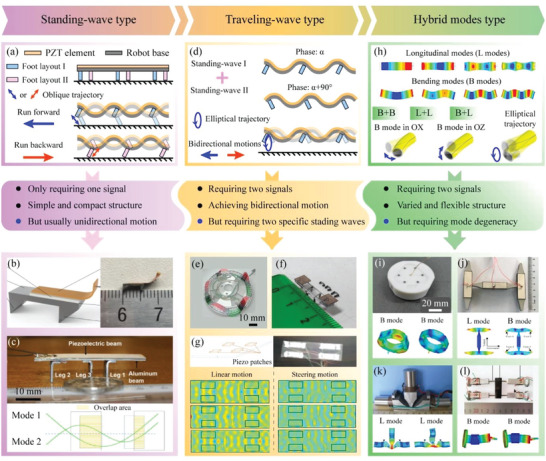
Working principles, features, and examples of the resonant type MPRs. a) Working principle of the standing‐wave type MPRs. b) A standing‐wave type biped MPR. Reproduced with permission.^[^ ^152^ ^]^ Copyright 2019, IEEE. c) A standing‐wave tripodal MPR. Reproduced with permission.^[^ ^153^ ^]^ Copyright 2017, IEEE. d) Working principle of the traveling‐wave type MPRs. e) A traveling‐wave type MPR with a ring structure. Reproduced with permission.^[^ ^156^ ^]^ Copyright 2020, IEEE. f) A traveling‐wave biped MPR. Reproduced with permission.^[^ ^157^ ^]^ Copyright 2020, MDPI. g) A traveling‐wave plate MPR. Reproduced with permission.^[^ ^159^ ^]^ Copyright 2018, IEEE. h) Working principle of the hybrid modes type MPRs. i) A hybrid mode type MPR with a ring structure. Reproduced with permission.^[^ ^162^ ^]^ Copyright 2022, IEEE. j) A hybrid mode type quadrupedal MPR. Reproduced with permission.^[^ ^163^ ^]^ Copyright 2018, IEEE. k) A hybrid mode type MPR with a T‐shaped structure. Reproduced with permission.^[^ ^164^ ^]^ Copyright 2016, Taylor & Francis. l) A quadrupedal MPR driven by bending‐bending hybrid mode. Reproduced with permission.^[^ ^165^ ^]^ Copyright 2018, MDPI.

The resonant type MPRs are featured by that their piezoelectric actuating elements work at the resonant modes, and can be further divided into the standing‐wave type ones, the traveling‐wave type ones, and the hybrid‐type ones according to the adopted vibration modes of the piezoelectric actuating elements,^[^ ^147^, ^148^, ^149^ ^]^ as illustrated in **Figure** **6**.

The standing‐wave type MPRs utilize the standing‐wave vibration modes of their piezoelectric elements to achieve movements, as shown in Figure 6a. In detail, a sinusoidal signal with the resonant frequency is applied to the piezoelectric ceramics to excite the standing‐wave vibration of the piezoelectric actuating element; and the driving feet are arranged at specific positions of the standing wave to obtain oblique trajectories with actuating function; then, movements of MPRs can be realized via the friction coupling effect between the driving feet and the ground.^[^ ^150^, ^151^ ^]^ In addition, the driving feet can be arranged at different positions of the standing wave to achieve the forward and backward motions, respectively. The standing‐wave type MPRs generally exhibited the advantages of simple and compact structures, requiring only one signal, and easy to excitation and control. Peng et al.^[^ ^152^ ^]^ proposed a standing‐wave type biped MPR, as illustrated in Figure 6b; the biped MPR was actuated by the second‐order bending vibration and could run forward with a maximum speed of 200 mm ^−1^ s. Generally, the standing‐wave type MPRs can only achieve unidirectional motion because the direction of oblique trajectory determined by the arrangement positions of the driving feet on the standing wave is constant. One common method to solve the limitation of unidirectional motion is using different vibration modes of one piezoelectric actuating element to obtain different directional oblique trajectory at the driving foot. As shown in Figure 6c, Hariri et al.^[^ ^153^ ^]^ presented a standing‐wave tripodal MPR, which could realize forward and backward motions by using the 1st‐order and 2nd‐order bending vibration modes, respectively.

The traveling‐wave type MPRs are actuated by the traveling‐wave vibration modes of their piezoelectric actuating elements. As illustrated in Figure 6d, the traveling‐wave vibration is composed of two standing‐wave vibrations with a 1/4 wavelength difference in space, a 90° phase difference in time and the same vibration mode, and an elliptical trajectory can be obtained at the driving feet to achieve the movements.^[^ ^154^, ^155^ ^]^ Unlike the constant oblique trajectory obtained by standing‐wave vibration, the traveling‐wave type MPRs can change the elliptical trajectory direction by exchanging the phase difference of the two standing waves to realize the bidirectional motions. Ma et al.^[^ ^156^ ^]^ designed a ring‐shaped and boundary fixed type MPR, as shown in Figure 6e; a rotary traveling wave was obtained by using two fifth‐order axial bending standing waves of the ring base, and the bi‐directional rotary motions were realized. However, for a long beam or thin rod structure with finite length, there are wave reflection at its both ends, which makes it impossible to obtain a linear traveling wave. In order to achieve linear motions of the traveling‐wave MPRs, García et al.^[^ ^157^, ^158^ ^]^ presented a MPR with a plate base and two piezoelectric patches, as shown in Figure 6f; the two piezoelectric patches were excited by two signals with a phase difference of 90° and the same frequency, in which the frequency was between the resonant frequencies of two contiguous bending modes; then, an approximate traveling wave was generated on the plate base to make the MPR run bidirectionally. Moreover, Hariri et al.^[^ ^159^ ^]^ proposed an MPR with a thin plate and four piezoelectric patches, as shown in Figure 6g, and two linear traveling waves could be generated on the thin plate to achieve linear and steering motions of robot.

The hybrid‐mode type MPRs are actuated by the hybrid vibration modes of their piezoelectric actuating elements. Similar to the traveling‐wave composed of two standing waves, the hybrid vibration modes are also obtained by superposing two standing‐wave vibration modes; and the difference is that the conditions of 1/4 wavelength difference in space and the same vibration modes are not required for the obtaining the hybrid vibration modes. As shown in Figure 6h, the standing‐wave modes used for MPRs are usually longitudinal modes (L modes) and bending modes (B modes); thus, the common hybrid modes are B–B, B–L, and L–L hybrid modes. Then, the elliptical or circular vibration trajectories can be generated at the driving feet by the hybrid vibration mode to make MPRs run forward, and the backward motions can be realized by exchanging the phase of the two standing‐wave vibration modes.^[^ ^160^, ^161^ ^]^ Liu et al.^[^ ^162^ ^]^ proposed a tripodal MPR with a ring‐shaped piezoelectric actuating element, as illustrated in Figure 6i; the robot was actuated by an axial‐radial bending hybrid vibration mode (B+B) of the ring base and could move linearly in three directions. Tian et al.^[^ ^163^ ^]^ presented a quadrupedal MPR with a H‐shaped structure, as shown in Figure 6j; the MPR utilized a longitudinal‐bending hybrid vibration mode (B+L), and could realize bidirectional linear motions. Moreover, Liu et al.^[^ ^164^ ^]^ designed a single‐legged MPR with a T‐shaped structure, as shown in Figure 6k; the robot was actuated by a longitudinal‐longitudinal hybrid vibration mode (L+L), and could run bidirectionally with a high speed more than 1 m s^−1^. Benefiting from diverse hybrid modes, structure designs of the hybrid‐mode type MPRs are very flexible and various; however, mode degeneracy is usually needed to achieve the same resonant frequency of the two different vibration modes, which require high accuracy for structural size. Fortunately, the resonant frequencies of two same‐order bending vibrations of symmetrical structures are the same; thus, the hybrid vibration mode of two same bending vibrations can be obtained without mode degeneracy, which is more popular for MPRs. For instance, Su et al.^[^ ^165^ ^]^ proposed a quadrupedal MPR, as shown in Figure 6l; which was driven by the B–B hybrid mode of a square section beam, and could achieve the linear, steering, and rotational motions.

In general, the resonant type MPRs exhibited high speed up to several hundreds of mm s^−1^ as their piezoelectric actuating elements work at the resonant modes and can generate relatively large deformations at the driving feet. For the three types of resonant MPRs, the standing‐wave type MPRs usually have simple and compact structures, and can utilize only one signal to realize movements; but the movements with the one signal are unidirectional. The traveling‐wave type MPRs also have simple structures and can realize bidirectional motions by two signals; but the traveling waves are easier obtained in axisymmetric structures to achieve rotational motions, the linear motions of MPRs require to excite approximate traveling waves of the long beam or thin rod structures. Then, the advantages of the hybrid‐mode type MPRs are various and flexible structure designs, but the mode degeneracy is needed for most of hybrid modes. Moreover, some resonant MPRs work at ultrasonic frequencies (>20 kHz) and have the advantage of no noise,^[^ ^166^, ^167^ ^]^ but there are usually wear and heat between the driving feet and ground, and the high‐speed motions are less controllable and irregular.

### 3.2 The Nonresonant Type MPRs

The piezoelectric actuating elements of non‐resonant type MPRs work in the low‐frequency ranges, which are usually much less than their 1st‐order resonant frequencies. The non‐resonant type MPRs include direct driving type MPRs, stepping actuating MPRs and inertial actuating MPRs, as plotted in Figure 5. Moreover, the direct driving type MPRs can be further divided into the direct deformation type ones and the combing flexure hinge type ones; the stepping driving type MPRs include the inchworm driving type ones and the walking driving type ones; and the inertial driving type MPRs can be divided into the inertial impact type ones and the stick‐slip actuating type ones.^[^ ^168^, ^169^, ^170^ ^]^

#### 3.2.1 The Direct Driving Type MPRs

**Figure 7:**
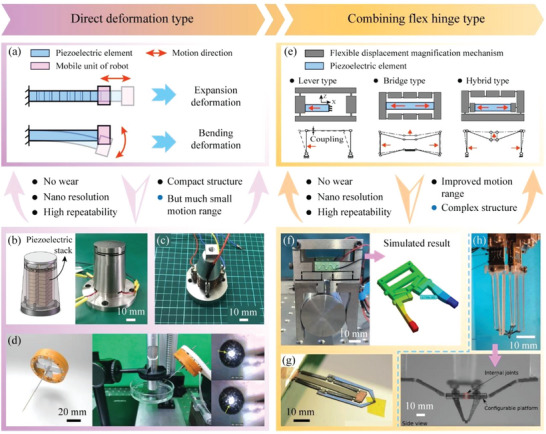
Working principles, features, and examples of the direct driving type MPRs. a) Working principle of the direct deformation type MPRs. b) A direct deformation type MPR driven by four piezoelectric stacks. Reproduced with permission.^[^ ^173^ ^]^ Copyright 2021, Elsevier. c) A direct deformation type MPR driven by a piezoelectric sandwich beam. Reproduced with permission.^[^ ^174^ ^]^ Copyright 2021, Elsevier. d) A 3‐DOF direct deformation type MPR driven by four piezoelectric patch beams. Reproduced with permission.^[^ ^175^ ^]^ Copyright 2023, IEEE. e) Working principle of the combining flexible hinge type MPRs. f) A crab‐like clamp MPR with a hybrid type FDAM. Reproduced with permission.^[^ ^180^ ^]^ Copyright 2022, IEEE. g) A direct deformation type MPR driven by four piezoelectric stacks. Reproduced with permission.^[^ ^181^ ^]^ Copyright 2017, IOP Publishing. h) A clamp MPR with a lever type FDAM.^[^ ^182^ ^]^ Copyright 2022, AAAS.

As shown in **Figure** **7**a, the direct deformation type MPRs take the deformations of their piezoelectric elements as the motion outputs of their mobile units directly, including elongation deformation of the piezoelectric stack and bending deformations of the piezoelectric tube, piezoelectric patch and sandwich beams.^[^ ^171^, ^172^ ^]^ Chang et al.^[^ ^173^ ^]^ proposed a direct deformation type MPR, as shown in Figure 7b; the mobile unit was actuated by four piezoelectric stacks to achieve deflecting motions in two orthogonal directions. Su et al.^[^ ^174^ ^]^ presented a micromanipulation MPR plotted in Figure 7c, which could achieve 3‐DOF motions on its top mobile unit by the direct deformations of a piezoelectric sandwich beam. Besides, Zhang et al.^[^ ^175^ ^]^ also designed a 3‐DOF micromanipulation MPR, as illustrated in Figure 7d; the 2‐DOF deflection motions and 1‐DOF linear motion of its mobile unit (a needle) were realized by cooperating the direct deformations of four piezoelectric bimorph beams with cross arrangement. The direct formation type MPRs have the advantages of no wear, high resolution (nano scale), high repeatability and compact structure; however, their motion ranges are determined by the deformations of the piezoelectric elements that are limited to tens of micrometers. In order to improve motion ranges of the direct deformation type MPRs, various flexible displacement amplification mechanisms (FDAMs) are combined with the piezoelectric actuating elements to amplify their direct deformations, as shown in Figure 7e, and the FDAMs can be divided into the lever type ones, the bridge type ones and the hybrid type ones.^[^ ^176^, ^177^, ^178^, ^179^ ^]^ Ma et al.^[^ ^180^ ^]^ proposed a crab‐like clamp MPR with a hybrid type FDAM, as illustrated in Figure 7f; the mobile unit was directly actuated by a piezoelectric stack, and the motion range was enlarged by nearly nine times by using the hybrid type FDAM. York et al.^[^ ^181^ ^]^ presented an ultra‐thin clamp MPR with a bridge type FDAM, as plotted in Figure 7g; the clamp was directly driven by a piezoelectric bimorph beam, and the clamp opening range was amplified to 106 µm. Moreover, Leveziel et al.^[^ ^182^ ^]^ designed a clamp MPR with a lever type FDAM, as shown in Figure 7h; the long rods were arranged at the end of the piezoelectric bimorph beams to amplify their bending displacements, and the flexible joints were used to convert the bending displacements into the opening and closing motions of the clamp unit. Indeed, the motion ranges of the direct drive type can be amplified by dozens of times by combining the FDAMs with the piezoelectric actuating elements, but the use of FDAMs also brings some shortcomings, including the relatively large and complex structure, the poor dynamic characteristics, and the existed coupling displacements.

#### 3.2.2 The Stepping Driving Type MPRs

**Figure 8:**
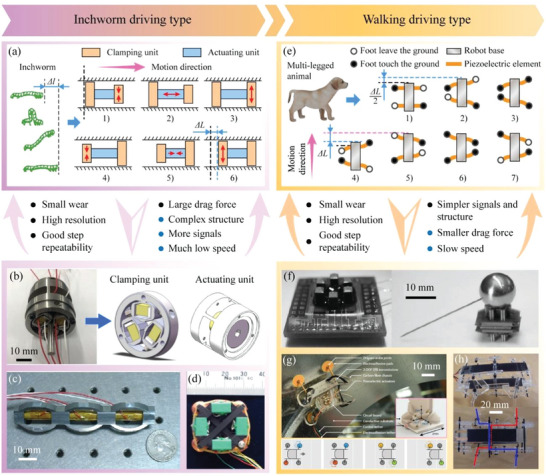
Working principles, features, and examples of the stepping driving type MPRs. a) Working principle of the inchworm driving type MPRs. b) An inchworm driving type MPR with two three‐jaw type clamping mechanism. Reproduced with permission.^[^ ^185^ ^]^ Copyright 2022, IOP Publishing. c) An inchworm MPR with three oval‐shaped shell structures. Reproduced with permission.^[^ ^186^ ^]^ Copyright 2005, IOP Publishing. d) A quadrupedal inchworm driving type MPR. Reproduced with permission.^[^ ^187^ ^]^ Copyright 2013, Elsevier. e) Working principle of the walking driving type MPRs. f) A walking driving type MPR with six legs. Reproduced with permission.^[^ ^190^ ^]^ Copyright 2002, IOP Publishing. g) A walking driving type MPR with electro‐adhesive pads and passive alignment ankles. Reproduced with permission.^[^ ^191^ ^]^ Copyright 2018, AAAS. h) A quadrupedal walking driving type MPR with two piezoelectric patch beams. Reproduced with permission.^[^ ^192^ ^]^ Copyright 2007, IEEE.

The stepping driving type MPRs are inspired by the movement of natural animals and achieve large‐range movements by accumulating small steps, and they can be divided into the inchworm driving type MPRs and the walking driving type MPRs according to the referenced animal motions. As shown in **Figure** **8**a, the inchworm driving type MPRs, inspired by the crawl motion of inchworm, usually have three groups of piezoelectric actuating elements (two clamping units and an actuating unit) to perform clamping and actuating motions, respectively; then, periodic stepping movements are achieved through the coordination of the clamping and actuating units.^[^ ^183^, ^184^ ^]^ Deng et al.^[^ ^185^ ^]^ designed an inchworm driving type MPR shown in Figure 8b, a three‐jaw type clamping mechanism including three piezoelectric stacks was developed to perform the clamping motion, and a linear movement was realized. Kim and Lee^[^ ^186^ ^]^ proposed an inchworm MPR with three unique oval‐shaped shell structures, as shown in Figure 8c; each shell structure was driven by a piezoelectric stack and could play the roles of clamping and actuating motions. Besides, Fuchiwaki^[^ ^187^ ^]^ presented a quadrupedal inchworm MPR plotted in Figure 8d, in which four electromagnet units and four piezoelectric stacks were used to perform the clamping and actuating motions, respectively. In fact, there is always static friction between the driving foot and the ground during the whole movement process, the inchworm driving type MPRs usually exhibit the advantages of small wear, high resolution, good step repeatability; besides, large drag forces can be obtained by the clamping units. However, the inchworm actuating method require MPRs to be deigned with more piezoelectric actuating elements, leading to the shortcomings of complex structure, requiring more signals; moreover, the speed of the inchworm driving type MPR is much slower due to the requirement to complete multiple stages in one step movement.

As shown in Figure 8e, the walking driving type MPRs are inspired by the multi‐legged animals and achieve movements by the coordination of their multiple driving feet. Generally, each driving foot have 2‐DOF deformations to perform the lifting and swinging motions, respectively; the number of driving feet is set as an even number of 4, 6, and 8, and they are divided into two groups to drive the robot alternately.^[^ ^188^, ^189^ ^]^ As illustrated in Figure 8f, Simu and Johansson^[^ ^190^ ^]^ proposed a walking driving type MPR with six legs, each leg was designed with a piezoelectric actuating element to achieve the lifting and swinging motions, and the robot could perform plane 3‐DOF movement with a tripod gait. Rivaz et al.^[^ ^191^ ^]^ presented a quadrupedal MPR using the walking actuating method, as illustrated in Figure 8g, which performed good climb ability by designing an electro‐adhesive pad and a passive alignment ankle for each leg. Moreover, in order to reduce the number of the used piezoelectric actuating elements, Lee et al.^[^ ^192^, ^193^ ^]^ proposed a quadrupedal MPR shown in Figure 8h; each leg was designed with a hip joint, and the rear legs and front legs were set as different height; thus, the lifting and swinging motions of the four legs could be realized by only two piezoelectric patch beams. Similar to the inchworm driving type MPRs, there is also always static friction between the driving foot and the ground for the walking driving type MPRs, which also exhibit the features of small wear, high resolution, and good step repeatability; besides, the walking driving type MPRs have no clamping units, thus, their structures and required signals are simpler, but the realized drag forces are reduced.

#### 3.2.3 The Inertial Driving Type MPRs

**Figure 9:**
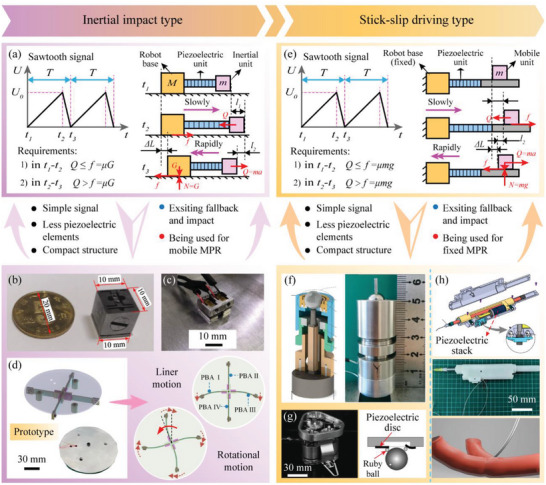
Working principles, features, and examples of the inertial driving type MPRs. a) Working principle of the inertial impact type MPRs. b) A cubic centimeter MPR using the inertial impact method. Reproduced with permission.^[^ ^199^ ^]^ Copyright 2019, Springer Nature. c) A bipedal inertial impact type MPR. Reproduced with permission.^[^ ^200^ ^]^ Copyright 2021, Springer Nature. d) A tripodal inertial impact type MPR actuated by four piezoelectric bimorph beams. Reproduced with permission.^[^ ^201^ ^]^ Copyright 2022, IOP Publishing. e) Working principle of the stick‐slip actuating type MPRs. f) A stick‐slip actuating type MPR capable of realizing rotary‐rotary motions. Reproduced with permission.^[^ ^202^ ^]^ Copyright 2019, IEEE. g) A tripodal MPR utilizing the slip‐stick method. Reproduced with permission.^[^ ^203^ ^]^ Copyright 2003, IEEE. h) A stick‐slip actuating type MPR capable of linear and rotary motions. Reproduced with permission.^[^ ^204^ ^]^ Copyright 2022, IEEE.

The inertial driving type MPRs make full use of the characteristics of fast response of piezoelectric materials and realize movements based on the principle of inertia, which can be divided into the inertial impact driving type MPRs and the stick‐slip MPRs according to whether the robots move as a whole.^[^ ^194^, ^195^, ^196^ ^]^ As shown in **Figure** **9**a, the inertial impact type MPRs usually have three parts, including robot base, piezoelectric unit, and inertial unit. Sawtooth signals are generally utilized to realize the inertial movements, and there are usually two sub‐steps in the movements: 1) in *t*_1_ to *t*_2_, the piezoelectric unit slowly deforms to push the inertial unit forward, the generated inertial force is smaller than the static friction between the robot base and the ground, and the robot base remains stationary; 2) in *t*_1_ to *t*_2_, the piezoelectric unit rapidly deforms and makes the piezoelectric unit move backward, the generated inertial force is much larger than that in sub‐step 1) because of the fast response; and the robot base will move forward when the inertial force generated by the inertial unit is greater than the friction force between the robot base and the ground; and the continuous movements can be achieved by repeating these sub‐steps^[^ ^197^, ^198^ ^]^ Zhong et al.^[^ ^199^ ^]^ proposed a cubic centimeter MPR using the inertial impact method, as shown in Figure 9b, only the forward movement was realized by using one piezoelectric stack. Then, Zhong et al.^[^ ^200^ ^]^ designed a bipedal inertial impact type MPR illustrated in Figure 9c to improve the freedom of the robot motion, two piezoelectric stacks were used to actuate the two driving feet, respectively, the forward and steering motions were achieved. Moreover, in order to further extend the motion DOF, Li et al.^[^ ^201^ ^]^ presented a tripodal inertial impact type MPR actuated by four piezoelectric bimorph beams with cross arrangement, as shown in Figure 9d, in which the bimorph beams were vertical to the ground were equipped with inertial units at their ends; through the cooperation of these four inertial units, the tripodal MPR could achieve plane 3‐DOF movements. Generally, the inertial impact type MPRs exhibit the advantages of simple and compact structures, requiring less piezoelectric actuating elements and signals, and faster speed than the stepping actuating MPRs; however, there are inherent problems of rollback motion of the inertial unit and small drag force.

Similar to the inertial impact type MPRs, the stick‐slip actuating type MPRs also include the three parts of robot base (fixed), piezoelectric unit, and mobile unit (inertial unit), and are excited by sawtooth signals, as shown in Figure 9e. The differences are that the robot base of the stick‐slip actuating type MPR is fixed, the relative sliding occurs between the piezoelectric element and the mobile unit, thus, only the mobile unit can move continuously. Gao et al.^[^ ^202^ ^]^ proposed a stick‐slip actuating type MPR, as illustrated in Figure 9f, which could realize 2‐DOF rotary‐rotary motions of the mobile unit (spherical joint) by the “slow‐fast” periodic deformation of the piezoelectric actuating element. Kortschack et al.^[^ ^203^ ^]^ designed a tripodal MPR driven by the slip‐stick method, as shown in Figure 9g; three spheres were used as the driving feet and were driven by piezoelectric discs to roll over the working surface, which was less damaging to the surface. Deng et al.^[^ ^204^ ^]^ presented a puncture MPR used the stick‐slip actuating method, as shown in Figure 9h; the piezoelectric unit was designed with a piezoelectric stack and a piezoelectric patch beam, which could actuate the mobile unit to perform linear and rotary motions, respectively. Usually, the stick‐slip actuating MPRs have the same characteristics as the inertial impact ones, the difference is that the stick‐slip actuating MPRs only actuate their mobile units and are designed as boundary fixed type MPRs. However, both the inertial impact MPRs and the stick‐slip actuating MPRs have the problems of rollback motion and small drag force. To deal with these problems, the inertial actuating MPRs driven by multiple legs alternately were proposed^[^ ^205^, ^206^, ^207^ ^]^; when one group of legs were in a relative sliding state with the ground or the mobile unit, the other group of legs was in a relative static state, the static friction force generated by the latter leg was larger than the sliding friction of the former leg; thus, the rollback motion could be suppressed, and the drag force could be improved. Besides, another method was to control the friction between the robot base and the ground or between the piezoelectric and mobile units by dynamically adjusting the positive pressure,^[^ ^208^, ^209^, ^210^ ^]^ the rollback motions during the relative sliding state could be reduced by increasing the positive pressure, and the drag force could be also improved.

### 3.3 MPRs Combining Different Working Principles

The characteristics of the above MPRs with different working principles are listed and compared in **Table** **2**, in which the items include size, weight, speed, agility, resolution (minimum step), load capacity, number of piezoelectric elements, and so on. Some possible advantages and disadvantages of MPRs with different working principles are summarized in **Table** **3**. Qualitatively speaking, the resonant type MPRs usually have the advantages of compact and flexible structure design, high speed up to several hundreds of mm ^−1^ s, and large load capacity; however, there are problems of wear, easy heating, small drag force, and poor agility. Although the agility can be improved by increasing the number of legs, the piezoelectric actuating elements corresponding to the increased legs can cause complex structure and control scheme. The direct deformation type MPRs can achieve high resolution up to nano lever with simple structure and hold other advantages of no wear and large load capacity, but the motion range is limited to a few microns by the direct deformation of the piezoelectric actuating element. Although the motion range can be enlarged to hundreds of microns by combining the flexible displacement amplification mechanism, it also leads to complex structure, reduced load capacity, and poor dynamic performance. Both the inchworm driving type and walking driving type MPRs have the advantages of stiction drive, less wear, large drag force, can achieving sub‐micron resolution and speed of a few mm ^−1^ s; however, their multiple legs usually also cause the complex structure and control scheme. The inertial driving type MPRs have the advantages of simple structure and exciting scheme, and can perform speed up to dozens of mm ^−1^ s and submicron resolution; however, there are usually disadvantages of rollback motion and small drag force.

**Table 2:** Summary of performance results of MPRs with different working principles (Note: 1) the values with * were not explicitly published in the literatures, they were calculated to the best of our ability using the available data; 2) L, S, R, D represent the linear, steering, rotational, and deflecting motions, respectively; subscripts *x, y*, and *z* represent the three directions of Cartesian coordinates; and the subscript F represents that the robot can only move forward; 3) “Number” in the table means the number of the used piezoelectric actuating elements in MPR).

Working principle	Size [mm]	Weight [g]	Speed [mm ^−1^s]	Resolution	Range [µm]	Load or thrust force	Number	Agility (DOF)	Refs.
Standing‐wave	31 × 44 × 20	9.8	231.7	/	Unlimited	331.6 g	1	L	[150]
Standing‐wave	6 × 2 × 2	0.135	200	/	Unlimited	18 g	1	L_F_	[152]
Traveling‐wave	20 × 3.5 × 2.7	0.24	100	/	Unlimited	9.6 g	1	L	[157]
Traveling‐wave	20 × 3 × *2	0.25	>120	/	Unlimited	/	1	L	[158]
Hybrid mode (B+B)	Φ 67 × 27	94	231.6	250 nm	Unlimited	300 g	1	L	[162]
Hybrid mode (B+L)	100 × 75 × 7	51.6	1641	/	Unlimited	1.2 N	1	L	[163]
Hybrid mode (L+L)	113 × 58 × 7	28	719	/	Unlimited	3.5 N	1	L	[164]
Direct deformation	Φ 36 × 49.5	180	/	0.28 µrad	4.5 × 4.5 mrad	36.7 g	4	D*_x_* + D*_y_*	[173]
Direct deformation	Φ 60 × 92	610	/	4.8 nm	13 × 13 × 3	/	1	L*_x_* + L*_y_* + L*_z_*	[174]
Combining flex hinge	150 × 150 × 100	/	/	5 nm	173.3 × 179.3 × 17.5	/	3	L*_x_* + L*_y_* + L*_z_*	[176]
Combining flex hinge	30 × 41 × 41	43	/	5 µrad	2 × 2 mrad	250 g	2	D*_x_* + D*_y_*	[177]
Inchworm actuating	Φ 34 × 40	220	0.16	*200 nm	Unlimited	12.3 N	7	L	[185]
Inchworm actuating	35 × 35 × 25	37.5	7.9	830 nm	Unlimited	100 g	4	L*_x_* + L*_y_* + R*_z_*	[187]
Walking actuating	68 × 60 × 50	450	1.16	740 nm	Unlimited	10 kg	6	L*_x_* + L*_y_* + R*_z_*	[189]
Walking actuating	45 × *30 × *20	1.48	4.6	/	Unlimited	*0.03 N	6	L + S	[191]
Inertial impact	10 × 10 × 10	7	13.1	*798 nm	Unlimited	3.15 N	1	L_F_	[199]
Inertial impact	15 × 10 × 9.5	/	3.55	/	Unlimited	/	2	L + S	[200]
Stick‐slip actuating	*35 × *32 × 9	35	5.96	50 nm	Unlimited	300	1	L_F_	[217]
Stick‐slip actuating	Φ 22 × 59	227	0.17 rad s^‐1^	2.3 µrad	Unlimited	1.85 mN m	1	D*_x_* + D*_y_*	[128]

**Table 3:** Advantages and disadvantages of MPRs with different working principles (Note: The characteristics here are a qualitative summary of the common characteristics of this type MPR, and excluding some specially designed examples).

Working principle of MPRs	Advantages	Disadvantages
Resonant actuating	Simple and compact structure, flexible design, high speed, relatively large load capacity, and simple signal	Less controllable and irregular motion, wear and heat between the driving feet and ground, and relatively low resolution
Direct actuating	High displacement resolution up to nanometer, no wear and heat, and simple signal	Small motion range of tens of micron (larger motion range of hundreds of microns by combing flexible hinge, but accompanied with low stiffness and bandwidth, and small output force)
Stepping actuating	Small wear and heat, large output force, good step repeatability	Relatively complex structure and signals, slow speed of several mm ^−1^ s, and requiring multiple piezoelectric actuating elements
Inertial actuating	Relatively simple structure and signal, less piezoelectric actuating elements	Relatively slow speed of tens of mm ^−1^ s, displacement rollback and wear problem

**Figure 10:**
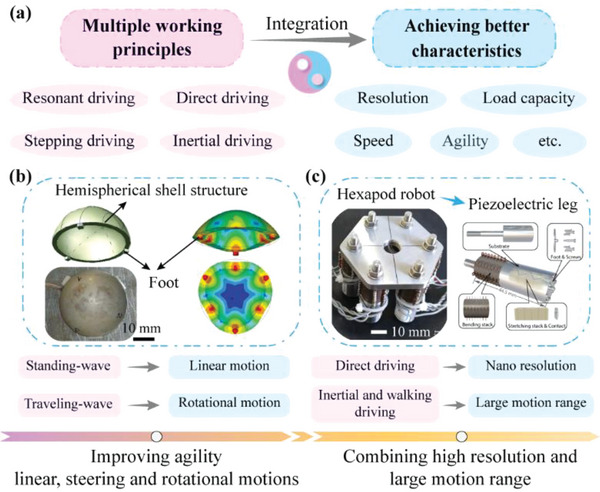
Schematic diagram of MPRs integrating different working principles. a) Integrating different working principles to achieve better characteristics. b) A resonant‐type MPR using both standing‐wave and traveling‐wave vibrations achieve the linear and the rotational motions. Reproduced with permission.^[^ ^213^ ^]^ Copyright 2013, SAGE Publications. c) A nonresonant type hexapod MPR utilizing the walking actuating and inertial impact, and direct deformation methods to achieve plane 3‐DOF motions with large range and nanoresolution. Reproduced with permission.^[^ ^214^ ^]^ Copyright 2021, Wiley‐VCH.

In general, MPRs with different working principles exhibit different advantages, and some of these advantages are contradictory for MPRs with only a single working principle, including the contradictories between the nano‐resolution and the large motion range, and between the simple structure, control, high agility, and so on. One interesting method to balance these contradictory features is to integrate multiple working principles into one MPR, as shown in **Figure** **10**a. It should be noted that the multiple working principles are conducted by the same piezoelectric actuating elements, rather than adding other piezoelectric actuating elements.^[^ ^211^, ^212^ ^]^ On the one hand, the motion agility can be improved without increasing extra structures by integrating different resonant operating principles. As illustrated in Figure 10b, Bansevicius et al.^[^ ^213^ ^]^ proposed a resonant type MPR with a hemispherical shell structure, whose standing‐wave and traveling‐wave vibrations were excited to achieve the linear and the rotational motions, respectively, the motion agility was successfully improved. On the other hand, the characteristics of nano‐resolution and large motion range can be both realized by integrating different non‐resonant working principles. Yu et al.^[^ ^214^ ^]^ presented a non‐resonant type hexapod MPR shown in Figure 10c, which could not only utilize the walking actuating and inertial impact methods to achieve plane 3‐DOF motions with large range, but also realize nano‐resolution of 5 nm by using the direct deformations of their legs.

## 4 Manufacturing Methods and Materials for MPRs

### 4.1 The Usual Manufacturing Methods for MPRs

**Figure 11:**
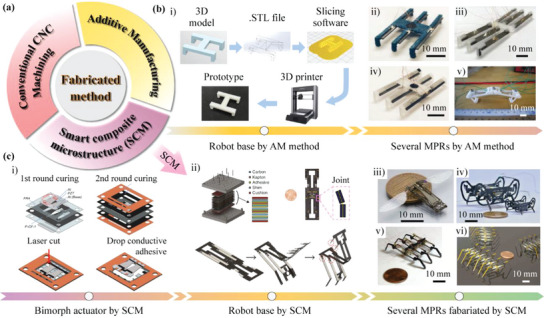
The new fabrication methods applied to MPRs to achieve miniature and special structure designs, mainly including additive manufacturing and smart composite microstructure. a) The common fabrication methods applied to MPRs. b) The common process and examples of AM method applied to MPRs: i) the usual main five steps in the 3D printing process; ii–iv) three hexapod MPR that using AM method to fabricate their robot bases. Reproduced with permission.^[^ ^229^ ^]^ Copyright 2018, IEEE; v) a biped MPR with a robot base fabricated by AM method. Reproduced with permission.^[^ ^231^ ^]^ Copyright 2018, ASME. c) The common process and examples of SCM method applied to MPRs: i) a piezoelectric patch beam fabricated by SCM method. Reproduced with permission.^[^ ^224^ ^]^ Copyright 2022, IEEE; ii) fabrication process of the robot base of an MPR using SCM method. Reproduced with permission.^[^ ^104^ ^]^ Copyright 2020, Springer Nature; iii) an aerial type MPR fabricated by SCM method. Reproduced with permission.^[^ ^242^ ^]^ Copyright 2012, IOP Publishing; iv) a quadrupedal MPR fabricated by SCM method. Reproduced with permission.^[^ ^243^ ^]^ Copyright 2020, IEEE; v) a hexapod MPR fabricated by SCM method. Reproduced with permission.^[^ ^244^ ^]^ Copyright 2011, Springer Nature; vi) an myriapod‐like MPR fabricated by SCM method. Reproduced with permission.^[^ ^245^ ^]^ Copyright 2012, IEEE.

Traditionally, the components of MPRs are manufactured by the CNC (computer numerical control) machining, and the robots can be fabricated by assembling the components and the piezoelectric actuating elements together with epoxy resin adhesive or bolts and nuts.^[^ ^215^, ^216^, ^217^, ^218^ ^]^ MPRs fabricated by the conventional CNC machining method usually use the metal material as the robot base, and have the advantages of high machining accuracy and high structural strength. However, the CNC machining method is difficult to process some special microstructures, which cannot meet the further miniaturization and special design requirements of MPRs. Then, some new fabrication methods are applied to MPRs with miniature and special structure designs, mainly including additive manufacturing (AM) method^[^ ^219^, ^220^, ^221^ ^]^ and smart composite microstructure (SCM) method,^[^ ^222^, ^223^, ^224^ ^]^ as shown in **Figure** **11**a. The AM method, also referred to as 3D printing method, has the unique advantages for the cost‐effective fabrication of complicated 3D geometries with multiple materials at multiple scales. As shown in i) of Figure 11b, the printing process usually includes five steps: 1) building the 3D model of the robot base by using 3D software; 2) converting the model to a file format that can be read by slicing software, such as STL. and OBJ., etc.; 3) dividing the 3D model into layer‐by‐layer sections by using the slicing software; 4) reading the slice file by the 3D printer; 5) printing out the desired 3D model. Moreover, there are many excellent reviews on AM, which introduce in detail its principles, classifications, and applications, etc..^[^ ^225^, ^226^, ^227^ ^]^ For MPRs fabricated by the AM method, their robot bases are usually designed to integrated structures and have the light weights. Oldham et al.^[^ ^228^, ^229^, ^230^ ^]^ designed a series of resonant MPRs, as shown in ii) to iv) of Figure 11b, in which the robot bases could be produced easily and quickly by the AM method, and MPRs were fabricated by pasting the piezoelectric sheets to the robot bases directly; these robots had body lengths of about 20 mm and weights of only 1 to 2 grams, and could run at speed of several hundreds of millimeters per seconds. Moreover, Dharmawan et al.^[^ ^231^ ^]^ proposed a two‐legged MPR, as shown in v) of Figure 11b, the components of the robot base were both produced by the AM method and assembled into a four‐bar linkage to drive the robot.

For SCM method, there are some other names, including Pop‐Up Book MEMS (micro electromechanical systems),^[^ ^232^, ^233^, ^234^ ^]^ printable robotics,^[^ ^235^, ^236^ ^]^ and lamina‐emergent mechanisms (LEM).^[^ ^237^, ^238^ ^]^ These different names have the common feature: materials are selectively added and removed layer by layer by utilizing batch material removal processes or rapid prototyping tools such as lasers, which can create various mechanical elements, such as structural elements, flexible hinges, and rigid connections; then, these mechanical elements can be used to form complex 3D geometries by folding or erecting their final shapes.^[^ ^239^, ^240^, ^241^ ^]^ For example, Liu et al.^[^ ^224^ ^]^ used SCM method to fabricate the piezoelectric bimorph beam, as shown in i) of Figure 11c. The layers of carbon fiber prepregs, piezoelectric ceramic, alumina ceramic, copper foil, and epoxy glass were laser machined with the designed patterns and pasted together after curing; then, the piezoelectric bimorph beam could be released from the frame by using laser cutting. Moreover, SCM methods could be also used to fabricate the robot base of MPRs. As shown in ii) of Figure 11c, Suzuki and Wood^[^ ^104^ ^]^ proposed a fixed‐type MPR, in which the robot base was a parallel mechanism fabricated by the pop‐up book MEMS technique; multiple layers with different materials were laser cut individually and laminated to create flexural joints and rigid links, and the parallelogram‐based mechanism could be assembled from a monolithic composite. As shown in iii) to vi) of Figure 11c, Wood et al. proposed a serial MRPs fabricated by SCM method, including the aerial type, the terrestrial type, and the boundary fixed type MPRs^[^ ^242^, ^243^, ^244^, ^245^ ^]^; these robots exhibited the advantages of light weight (only a few grams) and flexible structure design.

### 4.2 The Usual Piezoelectric Ceramic Materials used in MPRs

**Figure 12:**
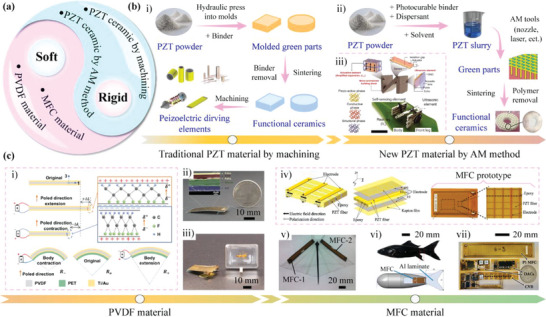
The common piezoelectric materials applied to MPRs. a) Classification of piezoelectric materials applied to MPRs. b) The rigid PZT ceramic materials applied to MPRs: i) The common fabrication process of traditional rigid PZT ceramic; ii) the common process of new rigid PZT ceramics fabricated by AM method; iii) a terrestrial MPR actuated by the new rigid PZT ceramics. Reproduced with permission.^[^ ^263^ ^]^ Copyright 2022, AAAS. c) The usual soft piezoelectric materials applied to MPRs: i) deformation principle of PVDF materials. Reproduced with permission.^[^ ^269^ ^]^ Copyright 2021, AAAS; ii) a bipedal MPR driven by one PVDF curved beam. Reproduced with permission.^[^ ^272^ ^]^ Copyright 2019, AAAS; iii) a tripodal MPR driven by two PVDF films. Reproduced with permission.^[^ ^269^ ^]^ Copyright 2021, AAAS; iv) the common structure of MFC laminate. Reproduced with permission.^[^ ^273^ ^]^ Copyright 2020, Elsevier; v) a soft underwater MPR driven by two MFC laminates. Reproduced with permission.^[^ ^279^ ^]^ Copyright 2011, IEEE; vi) a fish‐like underwater MPR driven by an MFC actuator. Reproduced with permission.^[^ ^280^ ^]^ Copyright 2021, IOP Publishing; vii) a soft terrestrial MPR driven by five MFC laminates connected together. Reproduced with permission.^[^ ^281^ ^]^ Copyright 2022, IEEE.

In addition to the manufacturing methods, the advance of the piezoelectric ceramic materials also promotes the development of MPRs. As shown in **Figure** **12**a, the piezoelectric ceramics that are applied in MPRs can be divided into the rigid ones and the soft ones, the former is usually prepared by two methods of traditional processing and AM, while the later mainly includes the PVDF (polyvinylidene difluoride) and MFC (macrofiber composite) materials.^[^ ^246^, ^247^, ^248^, ^249^, ^250^ ^]^ The rigid piezoelectric ceramics are usually made of PZT (lead zirconate titanate, an inorganic compound with the chemical formula Pb[Zr*_x_* Ti_1_−*x*]O_3(_0 ≤ *x* ≤1)), which has great sensitivity and high operating temperature^[^ ^251^, ^252^, ^253^ ^]^ The main steps of the traditional machining method to fabricate the PZT ceramics are shown in i) of Figure 12b, the PZT powders are made into PZT ceramics; then, the PZT ceramics are cut into the required shapes to manufacture the piezoelectric actuating elements.^[^ ^254^, ^255^ ^]^ This machining method for PZT ceramic is very mature, and most of MPRs utilize the rigid PZT ceramics fabricated by the traditional machining method. Another method to fabricate the PZT ceramics is based on AM, as shown in ii) of Figure 12b. The PZT powders are firstly made into PZT slurry by mixing with polymers and solutions in certain mixture ratios; then, the PZT ceramics can be fabricated by AM methods such as stereolithography, selective laser sintering, and fused deposition modeling.^[^ ^256^, ^257^, ^258^, ^259^ ^]^ The PZT element fabricated by AM method can achieve more nonzero and higher piezoelectric coefficients,^[^ ^260^, ^261^, ^262^ ^]^ which is beneficial to expand the vibration modes used by MPRs and improve the motion performance. For example, Cui et al.^[^ ^263^ ^]^ proposed a terrestrial MPR with length of about 16 mm and weight of 0.74 g, as shown in iii) of Figure 12b; the used PZT ceramics were fabricated by AM method and could be divided into different zones to separately serve as actuation element, self‐sensing element, and ultrasonic element, which could realize the actuation and sensing functions, respectively.

MPRs actuated by the rigid PZT ceramics can selectively achieve many advantages, such as fast response, large load capacity, high resolution, high speed, and so on. However, there are also some limitations to MPRs due to using the rigid PZT ceramics, mainly including the fragility of the ceramics and the small deformations of the piezoelectric actuating elements. The soft piezoelectric ceramic materials have the features of flexibility, light weight, and large deformation,^[^ ^264^, ^265^, ^266^ ^]^ which is conducive to the lightweight design and high adaptability of MPRs. One of the soft piezoelectric ceramic materials successfully applied to MPRs is PVDF, the corresponding structure of the piezoelectric actuating element and the deformation principle are shown in as shown in i) of Figure 12c. Similar to the d_31_ mode of the usual PZT ceramic sheet, the upper and lower surfaces of PVDF are plated with metal electrodes such as Ti/Au (titanium/gold) to facilitate the application of the exciting signals; and the PVDF film extends or contrasts along the horizontal direction when the applied electric field is in the opposite or the same direction of the polarization direction of the PVDF film.^[^ ^267^, ^268^, ^269^ ^]^ For MPRs, the structure of the piezoelectric actuating element based on the PVDF is usually the unimorph curved beam by adding a substrate layer such as PET (polyethylene terephthalate), in which the substrate layer does not change its length under the exciting signal; thus, the radius of the unimorph curved beam increases or decreases as the PVDF film is elongates or shortens.^[^ ^270^, ^271^ ^]^ Based on the deformation principle of the PVDF unimorph curved beam, Lin et al.^[^ ^272^ ^]^ proposed a bipedal MPR with length of 10 mm and weight of 0.024 g, as shown in ii) of Figure 12c, the robot could run at speed of 200 mm ^−1^ s and carry a load of 0.406 g; besides, the robot was crushed under a heavy load (59.5 kg), and could successfully operate after removing the heavy load, performing impressive robustness. Then, Lin et al.^[^ ^269^ ^]^ improved the original bipedal MPR driven by a single PVDF film into a tripodal MPR driven by two PVDF films, as shown in iii) of Figure 12c; the two front legs of the tripodal MPR were actuated by two PVDF films to achieve good agility and trajectory control. Another soft piezoelectric ceramic material successfully applied in MPRs is MFC. As shown in iv) of Figure 12c, the MFC is a laminate structure composed of piezoelectric fibers, epoxy matrix, copper electrodes, and Kapton film, in which the piezoelectric fibers usually have rectangular cross‐sections, and are interdigitated with the copper electrodes.^[^ ^273^, ^274^ ^]^ MFC usually works at the d_33_ mode, the polarization direction of the piezoelectric fiber is along its length direction, and the actuating voltage in d_33_ mode can reach up to 1500 V, which is beneficial to generate large deformation and output force.^[^ ^275^, ^276^ ^]^ Compared with the rigid PZT ceramics, the MFC laminates have the advantages of excellent structure flexibility, durability, large output force, and waterproof behavior, these advantages make the MFC laminates attract the favor of many scholars and be applied to MPRs, especially the underwater MPRs.^[^ ^277^, ^278^ ^]^ Zhao et al.^[^ ^279^ ^]^ proposed a soft underwater MPR mimicking a cow‐nosed ray, as shown in v) of Figure 12c; the robot used two pectoral fins separately actuated by two MFC laminates to swim under the water, and could realize a maximum speed of 200 mm ^−1^s at 10 Hz. As shown in vi) of Figure 12c, Meng et al.^[^ ^280^ ^]^ presented a fish‐like underwater MPR driven by the MFC actuator; two MFC laminates were pasted on the two sides of the caudal fin of the robot, and could realize a maximum thrust force of 7.35 mN under the water. Besides, Cheng et al.^[^ ^281^, ^282^ ^]^ proposed a soft terrestrial MPR driven by five MFC laminates connected together, as shown in vii) of Figure 12c; the robot adopted the inchworm actuating method and could achieve a step of 1.21 mm per cycle and carry a load of 200 g; moreover, the onboard power supply was integrated on each MFC laminate to realize the wireless motion.

## 5 Application Progresses of MPRs

**Figure 13:**
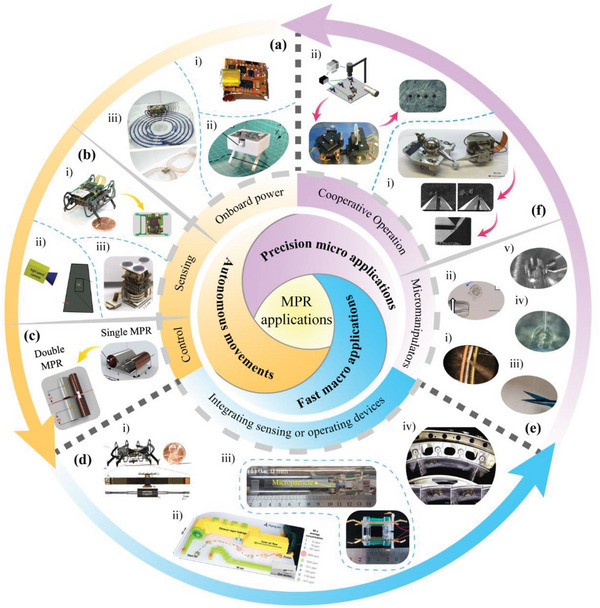
Application progresses of MPRs from the three aspects: autonomous movement of MPRs, applications of MPRs with fast macro motion, and applications of MPRs with precise micro motion. a) Examples of MPRs with integrated onboard power: i) an untethered resonant type MPR with a diamond‐shaped plate. Reproduced with permission.^[^ ^296^ ^]^ Copyright 2019, Wiley‐VCH.; ii) an inertial impact type MPR with an onboard power supply. Reproduced with permission.^[^ ^297^ ^]^ Copyright 2022, IOP Publishing; iii) an untethered MPR powered by wireless power delivery. Reproduced with permission.^[^ ^300^ ^]^ Copyright 2014, IEEE. b) Examples of MPRs with sensing integration: i) a power and control autonomous MPR with RF communication. Reproduced with permission.^[^ ^301^ ^]^ Copyright 2018, IEEE; ii) a motion capture system for MPR based on vision method. Reproduced with permission.^[^ ^303^ ^]^ Copyright 2017, IEEE; iii) a position sensing system for MPR based on the Moore effect. Reproduced with permission.^[^ ^305^ ^]^ Copyright 2006, IEEE. c) A soft MPR that using two PVDF films to control its motion trajectory. Reproduced with permission.^[^ ^306^ ^]^ Copyright 2023, Wiley‐VCH. d) Applications of MPRs with fast macro motion: i) handling small objects by equipping with a gripper. Reproduced with permission.^[^ ^313^ ^]^ Copyright 2020, IEEE. ii) executing the gas leakage detection mission with a gas sensor. Reproduced with permission.^[^ ^269^ ^]^ Copyright 2021, AAAS; iii) fine operation in a small pipe. Reproduced with permission.^[^ ^314^ ^]^ Copyright 2022, Springer Nature; iv) inspection of curved parts in a commercial jet engine. Reproduced with permission.^[^ ^191^ ^]^ Copyright 2018, AAAS. e) Applications of MPRs with precise micro motion: i) puncturing a vessel phantom with a microneedle. Reproduced with permission.^[^ ^104^ ^]^ Copyright 2020, Springer Nature; ii) realizing in situ three‐axial rotation of cells by the steady streaming generated around the oscillating pipette. Reproduced with permission.^[^ ^325^ ^]^ Copyright 2018, Springer; iii) cutting copper wires by equipping with a pair of microsurgical scissors. Reproduced with permission.^[^ ^326^ ^]^ Copyright 2023, AAAS; iv) performing the microinjection process of a zebrafish embryo by installing a glass microneedle. Reproduced with permission.^[^ ^326^ ^]^ Copyright 2021, Elsevier; v) assembling a wheel of a planetary micro gear by using a micro‐gripper. Reproduced with permission.^[^ ^328^ ^]^ Copyright 2000, SPIE. f) Coordinated operation of multiple MPRs with precise micro motion: i) grasping and releasing a pollen grain by the collaboration of two MPRs. Reproduced with permission.^[^ ^341^ ^]^ Copyright 2001, SPIE; ii) pulling out a leg from a minuscule insect through collaborative operation of three MPRs. Reproduced with permission.^[^ ^342^ ^]^ Copyright 2006, IEEE.

With the rapid development of various MPRs, MPRs have exhibited many impressive performances such as small size, high speed, fast response, high resolution, and so on. Then, the potential applications of MPRs are widely investigated and studied in numerous fields, and some of them have been commercialized.^[^ ^105^, ^283^, ^284^, ^285^ ^]^ In this section, we will introduce the application progresses of MPRs from the following three aspects: autonomous movement of MPRs, applications of MPRs with fast macro motion, and applications of MPRs with precise micro motion, as shown in **Figure** **13**.

The autonomous movement is an important foundation for MPRs to achieve practical applications, especially for the mobile MPRs. The challenge for MPRs to realize autonomous movement is that integrating onboard power supply, sensing, and control with the constraints of the finite size and weight.^[^ ^286^, ^287^, ^288^ ^]^ The integrated onboard power supply can help MPRs get rid of the interference and restraint of the wires to the movement, improving the straightness and range of motion. Unlike the traditional miniature robots driven by electromagnetic motors, which usually utilize simple DC (direct current) or PWM (pulse width modulation) signals,^[^ ^289^, ^290^ ^]^ the power supplies for MPRs need to be specially designed to generate the required exciting signals, mainly including the ramp signal for the direct driving type MPRs, the improved trapezoidal signal for the stepping driving type MPRs, the sawtooth signal for the inertial driving type MPRs, and the sine signal for the resonant type MPRs^[^ ^291^, ^292^, ^293^ ^]^; in addition, most of the exciting signals are need to be boosted to reach the voltage amplitude required by the piezoelectric actuating element.^[^ ^294^, ^295^ ^]^ For example, Mu et al.^[^ ^296^ ^]^ proposed an untethered resonant type MPR with size of 20 × 20 × 10 mm^3^ and weight of 1.815 g, as shown in i) of Figure 13a; the integrated power supply could output a square wave signal with a frequency of 30 Hz and voltage up to 240 V, and could power the robot to achieve a maximum speed of 20 mm ^−1^s. Wang et al.^[^ ^297^ ^]^ presented an inertial impact type MPR with an onboard power supply, as shown in ii) of Figure 13a; the power supply was limited with size of 35 × 28 × 56 mm^3^ and weight of 10.1 g, and could generate a sawtooth signal with frequency bandwidth from 0 to 1 kHz and voltage range from 0 to 30 V to power the robot. Besides, wireless power delivery is another effective method for MPRs to overcome the limitations of the wire traction problem.^[^ ^298^, ^299^ ^]^ As shown in iii) of Figure 13a, Karpelson et al.^[^ ^300^ ^]^ designed a wireless power transmission system based on magnetically coupled resonance to power their quadrupedal MPR; this system could wirelessly power the robot to run at a speed of 20 mm ^−1^ s. The wireless power delivery method can help MPRs overcome the restriction of onboard power supply due to the limited load capacity, but it requires external auxiliary devices such as coils and light source. In addition to integrating onboard power supply to achieve wireless movements, sensing and control integration are also important for the autonomous movements of MPRs. Goldberg et al.^[^ ^301^, ^302^ ^]^ proposed a power and control autonomous MPR with RF (radio frequency) communication, as shown in i) of Figure 13b; a MEMS (micro electromechanical systems) inertial measurement unit was used to estimate angular velocity and provide feedback, and two custom micro‐controllers were utilized to control its eight piezoelectric actuating elements to achieve trajectory control. Besides, some MPRs use external sending devices to realize sensing functions. Goldberg et al.^[^ ^303^ ^]^ developed a custom motion capture system based on vision method, as shown in ii) of Figure 13b; the system could rapidly and accurately track the position of their MPR successfully. Estana et al.^[^ ^304^, ^305^ ^]^ presented a position sensing system for their MPR based on the Moore effect, as shown in iii) of Figure 13b, three Moore‐based marks were equipped on the top of the MPR, the resolution and turn‐round‐time of the system reached 1 and 0.2 s, respectively. For the control integration of MPRs, except for the integrated control units, MPRs also needed to have enough sufficient motion agility. For example, Chen et al.^[^ ^306^ ^]^ proposed a soft MPRs driven by PVDF films, and the single robot could only perform linear motion with uncontrollable trajectory; then, two single robots were assembled together to achieve both the linear and turning motions, providing the basis for trajectory control, as shown in Figure 13c.

The application scenarios of MPRs are rich and diverse since MPRs with different piezoelectric actuating elements and working principles focus on realizing different aspects of characteristics, such as high resolution up to nanometer^[^ ^307^, ^308^ ^]^ and high speed up to hundreds of millimeters per second.^[^ ^309^, ^310^ ^]^ Therefore, we try to sort out the applications of MPRs into two categories, including the applications with fast macro motion and the applications of with precise micro motion. The former is based on the characteristics of high speed, flexible movement, small size, light weight, etc.; and it can cooperate with some sensing and operating modules to complete some applications, mainly including reconnaissance, handling, detection, and other application scenarios that require large‐range and rapid movement.^[^ ^311^, ^312^ ^]^ For example, Abondance et al.^[^ ^313^ ^]^ designed a miniature gripper composed of two piezoelectric unimorph beams, and equipped it to their MPR, as shown in i) of Figure 13d; the MPR could handle some small objects weighting up to 2.8 g. Besides, Lin et al.^[^ ^269^ ^]^ added a commercial gas sensor to their soft MPR to execute a gas leakage detection mission, as shown in ii) of Figure 13d. Xing et al.^[^ ^314^ ^]^ proposed an inertial driving type MPR, and demonstrated its potential application for fine operation in small pipe, as shown in iii) of Figure 13d. Moreover, De Rivaz et al.^[^ ^191^ ^]^ presented a quadrupedal MPR with voltage‐controlled electroadhesion to realize the climbing on inverted and vertical surfaces, and indicated its potential application for in‐situ inspection of high‐value assets, such as a curved portion of a commercial jet engine, as shown in iv) of Figure 13d.

For the applications of MPRs with precise micro motion, MPRs are based on the characteristics of high resolution, fast response, flexible movement, small size, etc. and are equipped with various micro tools to complete different micromanipulation tasks, such as micro puncture, micro handling, micro scribing, micro gripping, microassembly, and so on^[^ ^315^, ^316^, ^317^, ^318^, ^319^ ^]^; in which the micro tools mainly include microneedles, microgrippers, and micropipettes, etc.,^[^ ^320^, ^321^, ^322^, ^323^, ^324^ ^]^ as shown in Figure 13e. For instance, Suzuki and Wood^[^ ^104^ ^]^ assembled a microneedle on their MPR to puncture a vessel phantom (silicone tube, about 200 µm thick), demonstrating the potential application in retinal vein cannulation, as shown in i) of Figure 13e. Besides, Fuchiwaki et al.^[^ ^325^ ^]^ designed a pipette on their MPR and used the steady streaming generated around the oscillating pipette to realize the in situ three‐axial rotation of cells, as shown in ii) of Figure 13e. Li et al.^[^ ^326^ ^]^ proposed a fixed type MPR equipped with a pair of microsurgical scissors, which could be used to cut copper wires, pork, beef slices, intestines, etc., and the displacement resolution of the scissors reached up to 40 nm, as shown in iii) of Figure 13e. Hu et al.^[^ ^327^ ^]^ installed a glass micro‐needle on their MPR and performed the microinjection process of a zebrafish embryo, in which the embryo had a diameter of about 500 µm, as shown in iv) of Figure 13e. Schmoeckel et al.^[^ ^328^ ^]^ proposed an inertial driving type MPR equipped with a micro gripper, which could assembly a 500 µm‐diameter wheel of a planetary micro gear, as shown in v) of Figure 13e. On the one hand, MPRs with precise micro motion can successfully complete various micro manipulations by equipping the appropriate micro tools.^[^ ^329^, ^330^, ^331^, ^332^, ^333^, ^334^ ^]^ On the other hand, multiple MPRs with precise micro motion can be equipped with different micro tools for coordinated operation.^[^ ^335^, ^336^, ^337^, ^338^, ^339^, ^340^ ^]^ For instance, Wörn et al.^[^ ^341^ ^]^ designed two inertial driving type MPRs with the microgripper, one MPR grasped a pollen grain of 100 µm and move it to a target location, and the other MPR could grasp an additional probe to help release the pollen grain, as shown in i) of Figure 13f. Moreover, Aoyama et al.^[^ ^342^ ^]^ presented a micromachining system performed by multiple inchworm driving type MPRs, as shown in ii) of Figure 13f; one MPR was assembled a sample holder to move the sample material, the other MPR was equipped a microdrill, and the two MPRs could collaborate to make through holes with diameter of 50 µm under the combination of global and local path control.

## 6 Outlook and Conclusion

**Figure 14:**
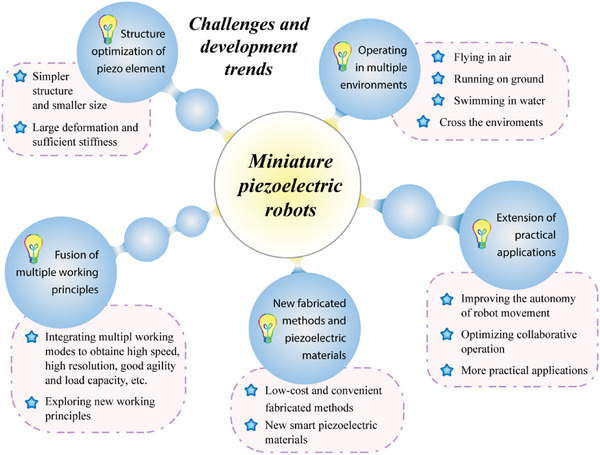
Challenges and development trends of MPRs from the aspects of operating environment, structure of piezoelectric actuating element, multiple working principles, new fabrication method and piezoelectric materials, and practical applications.

As mentioned above, many researchers have invested considerable effort into the research of MPRs in the past dozen years, which has greatly promoted the development of MPRs and contributed some impressive progress. In fact, the developed MPRs have successfully exhibited much demanding performance, such as body lengths as small as a few millimeters, body weights less than 1 g, high speed up to hundreds of millimeters per second, agile multi‐DOF movements, high resolution up to several nanometers, large load capacity up to several kilograms, etc. Therefore, comprehensively analyzing the existing research results, we discuss and evaluate some challenges and development trends in MPRs, as shown in **Figure** **14**, trying to provide some feasible research ideas for the further development of MPRs.

### 6.1 Structure Optimization of Piezoelectric Actuating Elements

As the core component of MPRs, the piezoelectric actuating elements mainly have four structural forms, including the piezoelectric stack, piezoelectric tube, piezoelectric patch beam, and piezoelectric sandwich beam. These piezoelectric actuating elements with different structural forms have different characteristics. For example, the piezoelectric patch beam has the simplest structure, which is conducive to the miniaturization of MPRs; but it usually has only 1‐DOF deformation output and low structural stiffness, which limits the agility and load capacity of MPRs. One interesting optimized structure is the piezoelectric composite beam with square cross section and ceramic sheets pasted on its four sides, named piezo‐leg,^[^ ^140^ ^]^ which combines the characteristics of simple structure, high stiffness, and 2‐DOF deformation output. Therefore, the optimization of the structure of the piezoelectric actuating element for achieving simpler structure and smaller size while ensuring large deformation and sufficient stiffness is an interesting research direction in MPRs.

### 6.2 Operating in Multiple Environments

MPRs can successfully operate in the different environments by combing the corresponding auxiliary structures, such as the foot, the microporous jet, and the flexible wing. Indeed, some researchers have developed several MPRs operating in multiple environments to further improve the environmental adaptability,^[^ ^113^, ^114^ ^]^ but there are still few related studies on MPRs operating in multiple environments, especially the transition in the different environments. Therefore, how to better realize the movement and transition of MPRs in multiple environments is another interesting research topic, which can help the MPRs improve the environment adaptability and expand application.

### 6.3 Fusion of Multiple Working Principle

MPRs have achieved many impressive performances, and these performances are realized by different MPRs based on different working principles, such as such as the high speed of the resonant type MPR, the nanometer resolution of the direct driving type MPR, and so on. However, these performances are contradictory for only a single working principle. For example, the resonant type MRP is usually easy to realize high speed due to its large vibration amplitude and high working frequency, but the high‐speed movement is usually irregular and less controllable, hindering the realization of high resolution. One useful and interesting method to combine these impressive performances is that integrating the multiple working modes into a single MPR, such as improving motion agility by using both the standing‐wave and the traveling‐wave actuating modes,^[^ ^213^ ^]^ and combining nanometer resolution and large motion range by utilizing both the direct actuating and the inertial actuating modes.^[^ ^214^ ^]^ Thus, how to make a single MPR integrate more working principles to obtain more excellent performances is a direction worth studying. Besides, exploring new working principle for MPRs is also a challenging and interesting research topic.

### 6.4 Attempts of New Fabricated Methods and Piezoelectric Materials

The application of the new fabricated method and new piezoelectric materials in MPRs have greatly promoted the development of MPRs. On the one hand, the SCM method, AM method, etc. can more conveniently make the special microstructures for MPRs.^[^ ^222^, ^223^, ^224^, ^228^, ^229^, ^230^, ^231^ ^]^ On the other hand, the new piezoelectric materials, especially the soft piezoelectric materials, have greatly expanded the performances of MPRs, mainly including the PVDF film that is conducive to the flexible body design and adaptability of MPRs,^[^ ^269^, ^272^ ^]^ and the MFC laminate that is suitable for the underwater MPRs due to its properties of large output force, waterproof, etc..^[^ ^273^, ^276^ ^]^ Performing the attempts of new manufacturing methods and piezoelectric materials in MPRs is valuable development trend, which is helpful for realizing the large‐scale and batch production of the customized microstructure and further improving the characteristics of MPRs.

### 6.5 Extension of Practical Applications

Thanks to the excellent characteristics of MPRs, their potential application scenarios are very rich and diverse, mainly including the fast macroapplications and the precision micro applications. However, except for some boundary fixed type MPRs that have been successfully commercialized,^[^ ^283^, ^284^, ^285^ ^]^ most of the application research stays in the experimental stage and has not been put into practice applications. The autonomous movement is an important foundation for MPRs, especially for the mobile MPRs, including the onboard power, sending, and control. Thus, improving the movement autonomy of MPRs is a meaning full research direction, which is conducive to the practice application. Besides, the collaborative operation of multiple MPRs is also an interesting topic, which is beneficial to expand the application scenarios, especially in the field of precision micromanipulation.

In summary, this paper mainly classifies and introduces MPRs from three aspects, including the operating environment, the structure of the used piezoelectric actuating element, and the working principle. Besides, the attempts of new fabricated methods and piezoelectric materials in MPRs are summarized, and application progresses of MPRs are analyzed and introduced. In a word, the aim of this paper is to make a comprehensive and insightful overview for the development of MPRs in the past dozen years. Notably, based on the summary and analysis of the development status of the MPRs, we discuss and conclude several challenges and development trends in terms of the structure of piezoelectric actuating element, the operating in multiple environments, fusion of multiple working principles, new manufacturing methods and piezoelectric material, and applications. We believe that the overview, discussion, and development trends of this review will greatly facilitate the further development of miniature piezoelectric robots.

## Conflict of Interest

The authors declare no conflict of interest.

## References

[1] D. Wang , Y. Liu , J. Deng , S. Zhang , J. Li , W. Wang , J. Liu , W. Chen , Q. Quan , G. Liu , H. Xie , J. Zhao , Adv. Sci. 2022, 9, e2203054. 10.1002/advs.202203054 PMC9561757 35981889

[2] Y. Wang , X. Du , H. Zhang , Q. Zou , J. Law , J. Yu , Adv. Sci. 2023, 10, e2207493. 10.1002/advs.202207493 PMC10288233 37097734

[3] Y. Chi , Y. Zhao , Y. Hong , Y. Li , J. Yin , Adv. Intell. Syst. 2023, 4, 2300063.

[4] C. Xu , Z. Yang , S. W. K. Tan , J. Li , G. Z. Lum , Adv. Intell. Syst. 2022, 4, 2100259.

[5] D. Kobo , B.‐E. Pinchasik , Adv. Intell. Syst. 2022, 4, 2200010.

[6] Y. Ozkan‐Aydin , D. I. Goldman , Sci. Rob. 2021, 6, eabf1628. 10.1126/scirobotics.abf1628 34321347

[7] R. Baines , S. K. Patiballa , J. Booth , L. Ramirez , T. Sipple , A. Garcia , F. Fish , R. Kramer‐Bottiglio , Nature 2022, 610, 283. 36224418 10.1038/s41586-022-05188-w

[8] J. Fan , Q. Du , Z. Dong , J. Zhao , T. Xu , Biomimetics 2022, 7, 142. 36278699 10.3390/biomimetics7040142 PMC9590051

[9] C. S. X. Ng , M. W. M. Tan , C. Xu , Z. Yang , P. S. Lee , G. Z. Lum , Adv. Mater. 2021, 33, 2003558. 10.1002/adma.202003558 33338296

[10] M. Sun , C. Tian , L. Mao , X. Meng , X. Shen , B. Hao , X. Wang , H. Xie , L. Zhang , Adv. Funct. Mater. 2022, 32, 2112508.

[11] Y. W. Lee , S. Chun , D. Son , X. Hu , M. Schneider , M. Sitti , Adv. Mater. 2022, 34, 2109325. 10.1002/adma.202109325 35060215

[12] Y. Tang , M. Li , T. Wang , X. Dong , W. Hu , M. Sitti , Adv. Mater. 2022, 34, 2204185. 10.1002/adma.202204185 PMC7613683 35975467

[13] C. Tang , B. Du , S. Jiang , Q. Shao , X. Dong , X. J. Liu , H. Zhao , Sci. Rob. 2022, 7, eabm8597. 10.1126/scirobotics.abm8597 35613300

[14] P. Bhushan , C. Tomlin , IEEE Rob. Autom. Lett. 2020, 5, 167.

[15] Q. Shi , Z. Gao , G. Jia , C. Li , Q. Huang , H. Ishii , A. Takanishi , T. Fukuda , IEEE Trans. Rob. 2021, 37, 747.

[16] Y. Zhang , R. Zhu , J. Wu , H. Wang , IEEE/ASME Trans. Mechatron. 2022, 27, 5748.

[17] C. H. Belke , J. Paik , IEEE/ASME Trans. Mechatron. 2017, 22, 2153.

[18] Q. Su , S. Zhang , Y. Liu , J. Deng , IEEE Trans. Ind. Electron. 2021, 68, 1466.

[19] Y. F. Zhang , C. J. X. Ng , Z. Chen , W. Zhang , S. Panjwani , K. Kowsari , H. Y. Yang , Q. Ge , Adv. Mater. Technol. 2019, 4, 1900427.

[20] Y. Lin , G. Yang , Y. Liang , C. Zhang , W. Wang , D. Qian , H. Yang , J. Zou , Adv. Funct. Mater. 2020, 30, 2000349.

[21] Z. Jiao , C. Ji , J. Zou , H. Yang , M. Pan , Adv. Mater. Technol. 2019, 4, 1800429.

[22] X. Huang , K. Kumar , M. K. Jawed , A. Mohammadi Nasab , Z. Ye , W. Shan , C. Majidi , Adv. Mater. Technol. 2019, 4, 1800540.

[23] H. T. Lee , F. Seichepine , G. Z. Yang , Adv. Funct. Mater. 2020, 30, 2070231.

[24] T.‐Y. Wu , Q.‐Y. Fang , Z.‐L. Xu , X.‐J. Li , W.‐X. Ma , M.‐S. Chu , J. H. Lim , K.‐C. Chuang , Adv. Intell. Syst. 2022, 4, 2200035.

[25] J. Sun , D. Zhou , Y. Liu , J. Deng , S. Zhang , IEEE Trans. Ind. Electron. 2023, 71, 1779.

[26] J. Sun , D. Zhou , J. Deng , Y. Liu , IEEE Trans. Ind. Electron. 2023, 70, 7153.

[27] D. Zhou , Y. Liu , X. Tang , J. Sun , J. Deng , IEEE Trans. Ind. Electron. 2022, 69, 11447.

[28] Z. Ren , S. Kim , X. Ji , W. Zhu , F. Niroui , J. Kong , Y. Chen , Adv. Mater. 2022, 34, 2106757. 10.1002/adma.202106757 34839551

[29] Y. Chen , H. Zhao , J. Mao , P. Chirarattananon , E. F. Helbling , N. P. Hyun , D. R. Clarke , R. J. Wood , Nature 2019, 575, 324. 31686057 10.1038/s41586-019-1737-7

[30] Y. Guo , L. Liu , Y. Liu , J. Leng , Adv. Intell. Syst. 2021, 3, 2000282.

[31] Y. Cao , J. Dong , IEEE/ASME Trans. Mechatron. 2021, 26, 854.

[32] S. Wu , Y. Hong , Y. Zhao , J. Yin , Y. Zhu , Sci. Adv. 2023, 9, eadf8014. 36947625 10.1126/sciadv.adf8014 PMC10032605

[33] Z. Yang , L. Zhang , Adv. Intell. Syst. 2020, 2, 2000082.

[34] J. Zhang , R. H. Soon , Z. Wei , W. Hu , M. Sitti , Adv. Sci. 2022, 9, e2203730. 10.1002/advs.202203730 PMC9631051 36065052

[35] J. Zhang , Y. Guo , W. Hu , M. Sitti , Adv. Mater. 2021, 33, 2100336. 10.1002/adma.202100336 PMC7612658 34048125

[36] T. Zhao , W. Fang , Y. Fan , Z. Hu , H. Wu , X. Q. Feng , J. a. Lv , Adv. Mater. Technol. 2022, 7, 2101660.

[37] X. Q. Wang , K. H. Chan , Y. Cheng , T. Ding , T. Li , S. Achavananthadith , S. Ahmet , J. S. Ho , G. W. Ho , Adv. Mater. 2020, 32, 2000351. 10.1002/adma.202000351 32285545

[38] M. Sitti , D. S. Wiersma , Adv. Mater. 2020, 32, 1906766. 10.1002/adma.201906766 32053227

[39] M. Xun , H. Yu , Y. Liu , J. Deng , S. Zhang , K. Li , IEEE/ASME Trans. Mechatron. 2023, 28, 223.

[40] H. Li , W. Chen , Y. Feng , J. Deng , Y. Liu , Int. J. Mech. Sci. 2023, 240, 107926.

[41] H. Li , J. Liu , Y. Feng , J. Deng , Y. Liu , IEEE Trans. Ind. Electron. 2023, 70, 9336.

[42] P. Du , L. Han , X. Qiu , W. Chen , J. Deng , Y. Liu , J. Zhang , Int. J. Mech. Sci. 2022, 222, 107239.

[43] Y. Feng , J. Liu , H. Li , X. Ma , P. Du , K. Li , Y. Liu , Int. J. Heat Mass Transfer 2022, 192, 122902.

[44] Q. Chang , X. Gao , Y. Liu , J. Deng , S. Zhang , W. Chen , Mech. Syst. Signal Process. 2022, 174, 109072.

[45] F. Lu , Y. Liu , W. Chen , J. Deng , K. Li , S. Zhang , X. Tian , Mech. Syst. Signal Process. 2022, 172, 109009.

[46] P. Du , Y. Liu , W. Chen , S. Zhang , J. Deng , IEEE Trans. Ultrason. Ferroelectr. Freq. Control 2021, 68, 2766. 33970860 10.1109/TUFFC.2021.3078663

[47] K. Li , Y. Liu , J. Liu , W. Chen , IEEE Trans. Ind. Electron. 2022, 69, 2728.

[48] J. Li , J. Deng , Y. Liu , H. Yu , X. Tian , K. Li , IEEE Trans. Ind. Electron. 2022, 69, 5091.

[49] J. Li , Y. Liu , J. Deng , S. Zhang , W. Chen , IEEE Trans. Ind. Electron. 2022, 69, 10407.

[50] H. Moradian , G. R. Vossoughi , Proc. Inst. Mech. Eng., Part C 2015, 230, 2413.

[51] S. Lin , J. Ma , J. Li , S. Li , M. Wang , Y. Hu , J. Wen , Int. J. Mech. Sci. 2023, 244, 108071.

[52] A. Ceponis , D. Mazeika , V. Jurenas , Micromachines 2021, 12, 1396.. 34832807 10.3390/mi12111396 PMC8625604

[53] X. Gao , Z. Li , J. Wu , X. Xin , X. Shen , X. Yuan , J. Yang , Z. Chu , S. Dong , Research 2019, 2019, 8232097. 31922139 10.34133/2019/8232097 PMC6946261

[54] R. Bansevicius , G. Kulvietis , V. Jurenas , J. Janutenaite‐Bogdaniene , J. Vibroeng. 2017, 19, 5182. 10.3390/mi9110597 PMC6266650 30445690

[55] M. Goldfarb , M. Gogola , G. Fischer , E. Garcia , J. Micromechatronics 2002, 1, 205.

[56] W. Wang , J. Deng , J. Li , S. Zhang , Y. Liu , IEEE Trans. Ind. Electron. 2023. 10.1109/tie.2023.32665711.

[57] A. Arbat , E. Edqvist , R. Casanova , J. Brufau , J. Canals , J. Samitier , S. Johansson , A. Diéguez , Sens. Actuators, A 2009, 153, 76.

[58] D. Kim , Z. Hao , A. R. Mohazab , A. Ansari , Int. J. Nonlinear Mech. 2020, 127, 103551.

[59] H. H. Hariri , G. S. Soh , S. Foong , K. L. Wood , in Int. Design Eng. Technical Conf. and Computers and Information in Engineering Conf. 2019 , Vol. 59247, ASME, New York 2019, V05BT07A004.

[60] P. Zhu , H. Peng , X. Lu , M. Guo , G. Zhao , W. Liu , Smart Mater. Struct. 2020, 29, 045009.

[61] H. Peng , P. Lu , J. Hu , L. Mao , K. Zhao , Smart Mater. Struct. 2020, 29, 015011.

[62] J. Zhou , M. Suzuki , R. Takahashi , K. Tanabe , Y. Nishiyama , H. Sugiuchi , Y. Maeda , O. Fuchiwaki , IEEE Rob. Autom. Lett. 2020, 5, 6717.

[63] O. Fuchiwaki , K. Arafuka , S. Omura , IEEE/ASME Trans. Mechatron. 2012, 17, 697.

[64] J. Li , J. Deng , S. Zhang , Y. Liu , Int. J. Mech. Sci. 2023, 250, 108276.

[65] H. Yu , J. Deng , Y. Liu , Y. Wang , Int. J. Mech. Sci. 2023, 240, 107943.

[66] S. A. Rios , A. J. Fleming , Y. K. Yong , IEEE/ASME Trans. Mechatron. 2018, 23, 524.

[67] J. Hu , S. Chen , L. Wang , IEEE Trans. Ind. Electron. 2023, 70, 8194. 10.1109/TIE.2022.3220864 PMC7614654 37323755

[68] L. Wang , W. Chen , J. Liu , J. Deng , Y. Liu , Mech. Syst. Signal Process. 2019, 133, 106254.

[69] X. Q. Tian , Y. X. Liu , J. Deng , L. Wang , W. S. Chen , Sens. Actuators, A 2020, 306, 111971.

[70] H. Li , J. Liu , K. Li , Y. Liu , Mech. Syst. Signal Process. 2021, 151, 107393.

[71] S. Mohith , A. R. Upadhya , K. P. Navin , S. M. Kulkarni , M. Rao , Smart Mater. Struct. 2020, 30, 013002.

[72] X. Gao , J. Yang , J. Wu , X. Xin , Z. Li , X. Yuan , X. Shen , S. Dong , Adv. Mater. Technol. 2019, 5, 1900716.

[73] J. Deng , C. Yang , Y. Liu , S. Zhang , J. Li , X. Ma , H. Xie , Sci. China: Technol. Sci. 2023, 66, 821.

[74] H. Jalili , G. Vossoughi , H. Salarieh , J. Comput. Nonlinear Dyn. 2016, 11, 021003.

[75] F. L. N‐Nagy , J. H. Calderwood , Int. J. Control 1969, 10, 529.

[76] G. C. Joyce , G. C. Wilson , J. Phys. E: Sci. Instrum. 1969, 2, 661. 10.1088/0022-3735/2/8/317 5807886

[77] J. G. Smits , Sens. Actuators, A 1992, 35, 129.

[78] A. Ferreira , P. Minotti , Rev. Sci. Instrum. 1997, 68, 1779.

[79] N. Lobontiu , M. Goldfarb , E. Garcia , Mech. Mach. Theory 2001, 36, 425.

[80] G. Muscato , Intell. Autom. Soft Comput. 2004, 10, 267.

[81] J. Zhao , G. Mu , H. Dong , T. Sun , K. T. V. Grattan , IEEE Trans. Ind. Electron. 2023, 70, 9260.

[82] F. Becker , K. Zimmermann , T. Volkova , V. T. Minchenya , Regul. Chaotic Dyn. 2013, 18, 63.

[83] N. T. Jafferis , E. F. Helbling , M. Karpelson , R. J. Wood , Nature 2019, 570, 491. 31243384 10.1038/s41586-019-1322-0

[84] D. Tan , Y.‐A. Le Dault , A. Erturk , A. Erturk , Proc. SPIE 2019, 10967, 109670X.

[85] P. Xiao , N. Yi , T. Zhang , Y. Huang , H. Chang , Y. Yang , Y. Zhou , Y. Chen , Adv. Sci. 2016, 3, 1500438. 10.1002/advs.201500438 PMC5071709 27818900

[86] X. Zhang , M. Lok , T. Tong , S. K. Lee , B. Reagen , S. Chaput , P.‐E. J. Duhamel , R. J. Wood , D. Brooks , G.‐Y. Wei , IEEE J. Solid‐State Circuits 2017, 52, 2374.

[87] A. T. Baisch , C. Heimlich , M. Karpelson , R. J. Wood , in 2011 IEEE/RSJ Int. Conf.on Intelligent Robots and Systems, IEEE, Piscataway, NJ 2011, p. 5073.

[88] H. Hida , Y. Morita , F. Kurokawa , Y. Tsujiura , I. Kanno , Microsyst. Technol. 2016, 22, 1429.

[89] A. Erturk , in Robot Fish: Bio‐inspired Fishlike Underwater Robots (Ed: R. Du ), Springer, Berlin 2015, p. 255.

[90] L. Cen , A. Erturk , Bioinspiration Biomimetics 2013, 8, 016006. 23348365 10.1088/1748-3182/8/1/016006

[91] X. Lu , Z. Wang , H. Shen , K. Zhao , T. Pan , D. Kong , J. Twiefel , Appl. Sci. 2019, 10, 31.

[92] K. Li , X. Zhou , Y. Liu , J. Sun , X. Tian , H. Zheng , L. Zhang , J. Deng , J. Liu , W. Chen , J. Zhao , Adv. Intell. Syst. 2023, 5, 2200262.

[93] L. Junqiang , Y. Yiling , W. Chuanyu , L. Guoping , C. Tehuan , M. Jianqiang , Sens. Actuators, A 2020, 303, 111587.

[94] X. Zhou , K. Li , Y. Liu , J. Sun , H. Li , W. Chen , J. Deng , IEEE Trans. Ind. Electron. 2023, 70, 5044.

[95] T. Wiguna , S. Heo , P. Hoon Cheol , G. Nam Seo , J. Intell. Mater. Syst. Struct. 2008, 20, 751.

[96] Q. Zhao , S. Liu , J. Chen , G. He , J. Di , L. Zhao , T. Su , M. Zhang , Z. Hou , Rob. Auton. Syst. 2021, 140, 103733.

[97] Y. Chen , Y. Liu , T. Liu , H. Li , S. Qu , W. Yang , Sci. China: Technol. Sci. 2022, 65, 1749.

[98] Y. Du , B. Peng , W. Zhou , Y. Wu , in 2022 IEEE 35th Int. Conf. on Micro Electro Mechanical Systems Conf. (MEMS), IEEE, Piscataway, NJ 2022, p. 644.

[99] S. Zhou , W. Zhang , Y. Zou , X. Ke , F. Cui , W. Liu , Electron. Lett. 2017, 53, 579.

[100] A. Zhang , L. Wang , J. Jin , D. Chen , R. Liu , H. Zhao , Smart Mater. Struct. 2021, 30, 105032.

[101] Z. Ye , C. Zhou , J. Jin , P. Yu , F. Wang , Ultrasonics 2019, 96, 90. 30833181 10.1016/j.ultras.2019.02.007

[102] Z. Du , R. Shi , W. Dong , IEEE Trans. Rob. 2014, 30, 131.

[103] Y. Liu , J. Deng , Q. Su , IEEE Access 2018, 6, 59986.

[104] H. Suzuki , R. J. Wood , Nat. Mach. Intell. 2020, 2, 437.

[105] Kleindiek Nanotechnik GmbH , Germany, https://www.nanotechnik.com/mm3e.html. (accessed: September 2023)

[106] H. McClintock , F. Z. Temel , N. Doshi , J. S. Koh , R. J. Wood , Sci. Rob. 2018, 3, eaar3018. 10.1126/scirobotics.aar3018 33141699

[107] C. Wang , W. Zhang , J. Zhao , J. Hu , Y. Zou , Nano Lett. 2020, 15, 1079.

[108] E. Farrell Helbling , R. J. Wood , Appl. Mech. Rev. 2018, 70, 010801.

[109] P. Chirarattananon , K. Y. Ma , R. J. Wood , Int. J. Rob. Res. 2016, 35, 1185.

[110] T. Ozaki , K. Hamaguchi , IEEE Rob. Autom. Lett. 2018, 3, 4217.

[111] K. Y. Ma , P. Chirarattananon , S. B. Fuller , R. J. Wood , Science 2013, 340, 603. 23641114 10.1126/science.1231806

[112] E. T. K. Chiang , T. Urakubo , T. Mashimo , IEEE Access 2022, 10, 13210.

[113] Y. Chen , N. Doshi , B. Goldberg , H. Wang , R. J. Wood , Nat. Commun. 2018, 9, 2495. 29950597 10.1038/s41467-018-04855-9 PMC6021446

[114] Y. Chen , H. Wang , E. F. Helbling , N. T. Jafferis , R. Zufferey , A. Ong , K. Ma , N. Gravish , P. Chirarattananon , M. Kovac , R. J. Wood , Sci. Rob. 2017, 2, eaao5619. 10.1126/scirobotics.aao5619 33157886

[115] T. Mashimo , T. Urakubo , Y. Shimizu , IEEE/ASME Trans. Mechatron. 2018, 23, 781.

[116] J. Xing , W. Jin , K. Yang , I. Howard , IEEE Trans. Ind. Electron. 2023, 70, 12596.

[117] H. Jalili , H. Salarieh , G. Vossoughi , Nonlinear Dyn. 2017, 89, 1927.

[118] A. Torii , M. Nishio , K. A. E. Doki , A. Ueda , Electr. Eng. Jpn. 2016, 196, 22.

[119] A. Torii , A. Ueda , K. Doki , Electr. Eng. Jpn. 2011, 177, 33.

[120] H. G. Kim , J. N. Kim , T. W. Na , K. C. Park , I. K. Oh , Adv. Mater. Technol. 2018, 3, 1800298.

[121] S. Yan , F. Zhang , Z. Qin , S. Wen , Smart Mater. Struct. 2006, 15, N7.

[122] A. Torii , Y. Mitsuyoshi , S. Mototani , K. Doki , Int. J. Autom. Technol. 2018, 12, 784.

[123] T. Takami , X. L. Deng , J. W. Son , B. H. Park , T. Kawai , Jpn. J. Appl. Phys. 2012, 51, 08KB12.

[124] D. S. Raghunvanshi , S. I. Moore , A. J. Fleming , Y. K. Yong , IEEE/ASME Trans. Mechatron. 2020, 25, 1479.

[125] B. J. Nelson , S. M. Martel , J.‐M. Breguet , I. W. Hunter , Proc. SPIE 2001, 4568, 199.

[126] S. Martel , Int. J. Rob. Res. 2005, 24, 575.

[127] B. J. Nelson , S. M. Martel , J.‐M. Breguet , L. Cervera Olague , J. Bautista Coves Ferrando , S. Riebel , T. Koker , J. Suurkivi , T. Fofonoff , M. Sherwood , R. Dyer , I. W. Hunter , Proc. SPIE 2001, 4568, 231.

[128] X. Gao , J. Deng , S. Zhang , J. Li , Y. Liu , IEEE Trans. Ind. Electron. 2022, 69, 3928.

[129] H. Hariri , Y. Bernard , A. Razek , J. Intell. Mater. Syst. Struct 2015, 26, 2577.

[130] G. Wang , C. Li , T. Yuan , Rev. Sci. Instrum. 2017, 88, 115001. 29195366 10.1063/1.4991063

[131] Y. Wang , B. Wang , Y. Zhang , L. Wei , C. Yu , Z. Wang , Z. Yang , Int. J. Mech. Sci. 2022, 231, 107596.

[132] S. A. Rios , A. J. Fleming , Y. K. Yong , IEEE Rob. Autom. Lett. 2017, 2, 337.

[133] X. Zhang , G. Zhang , K. Nakamura , S. Ueha , Sens. Actuators, A 2011, 169, 206.

[134] Q. Zhang , W. Chen , Y. Liu , J. Liu , Q. Jiang , IEEE Trans. Ind. Electron. 2017, 64, 2188.

[135] S. Zhang , J. Liu , J. Deng , Y. Liu , IEEE Trans. Ind. Electron. 2019, 66, 7861.

[136] J. Deng , Y. Liu , X. Tian , S. Zhang , Smart Mater. Struct. 2019, 28, 115010.

[137] J. Deng , Y. Liu , W. Chen , H. Yu , IEEE/ASME Trans. Mechatron. 2019, 24, 207.

[138] J. Deng , Y. Liu , J. Liu , D. Xu , Y. Wang , IEEE Trans. Ind. Electron. 2019, 66, 6141.

[139] O. Ozcan , A. T. Baisch , R. J. Wood , in 2013 IEEE/RSJ Int. Conf. on Intelligent Robots and Systems (IROS) (Ed: N. Amato ), IEEE, Piscataway, NJ 2013, p. 1438.

[140] Y. Liu , J. Li , J. Deng , S. Zhang , W. Chen , H. Xie , J. Zhao , Adv. Intell. Syst. 2021, 3, 2100015.

[141] V. Ruiz‐Diez , J. Hernando‐Garcia , J. Toledo , A. Ababneh , H. Seidel , J. L. Sanchez‐Rojas , Micromachines 2020, 11, 517. 32443680 10.3390/mi11050517 PMC7281763

[142] L. Wang , Y. Hou , K. Zhao , H. Shen , Z. Wang , C. Zhao , X. Lu , Sens. Actuators, A 2019, 295, 428.

[143] Y. Wang , J. Deng , S. Zhang , H. Li , W. Chen , Y. Liu , Int. J. Smart and Nano Mater. 2022, 13, 346.

[144] P. Liu , Z. Wen , L. Sun , Sci. Bull. 2009, 54, 2134.

[145] S. K. Cheon , M. H. Park , S. S. Jeong , H. I. Jun , T. H. Kim , T. G. Park , Integr. Ferroelectr. 2019, 195, 81.

[146] D. Xu , Y. Liu , J. Liu , S. Shi , W. Chen , IEEE Access 2016, 4, 2371.

[147] A. G. Dharmawan , H. H. Hariri , S. Foong , G. S. Soh , K. L. Wood , in 2017 IEEE Int. Conf. on Robotics and Automation (ICRA), IEEE, Piscataway, NJ 2017, p. 6008.

[148] A. Čeponis , V. Jūrėnas , D. Mažeika , IOP Conf. Ser.: Mater. Sci. Eng. 2022, 1239, 012015.

[149] A. Ceponis , D. Mazeika , V. Jurenas , D. Deltuviene , R. Bareikis , Micromachines 2022, 13, 1763. 36296116 10.3390/mi13101763 PMC9611161

[150] J. Li , J. Deng , S. Zhang , W. Wang , Y. Liu , IEEE/ASME Trans. Mechatron. 2023. 10.1109/tmech.2023.3269014.

[151] P. Fan , C. Li , J. Braz. Soc. Mech. Sci. Eng. 2019, 41, 539.

[152] H. Peng , J. Yang , X. Lu , P. Zhu , D. Wu , IEEE Trans. Ind. Electron. 2019, 66, 7852.

[153] H. H. Hariri , G. S. Soh , S. Foong , K. Wood , IEEE Trans. Rob. 2017, 33, 742.

[154] L. Wang , C. Shu , J. Jin , J. Zhang , Smart Mater. Struct. 2017, 26, 035003.

[155] H. Hariri , Y. Bernard , A. Razek , Smart Mater. Struct. 2014, 23, 025013.

[156] X. Ma , J. Liu , J. Deng , Q. Liu , Y. Liu , IEEE Trans. Ultrason. Ferroelectr. Freq. Control 2020, 67, 1462. 32054574 10.1109/TUFFC.2020.2972307

[157] J. Hernando‐Garcia , J. L. Garcia‐Caraballo , V. Ruiz‐Diez , J. L. Sanchez‐Rojas , Micromachines 2020, 11, 321. 32244877 10.3390/mi11030321 PMC7142472

[158] J. Hernando‐Garcia , J. L. Garcia‐Caraballo , V. Ruiz‐Diez , J. L. Sanchez‐Rojas , Micromachines 2021, 12, 171. 33572248 10.3390/mi12020171 PMC7915569

[159] H. Hariri , Y. Bernard , A. Razek , IEEE/ASME Trans. Mechatron. 2018, 23, 242.

[160] Y. X. Liu , J. P. Yan , L. Wang , W. S. Chen , IEEE Trans. Ind. Electron. 2019, 66, 3041.

[161] H. Li , Y. Liu , J. Deng , W. Chen , K. Li , Sens. Actuators, A 2021, 331, 113029.

[162] J. Li , J. Deng , Y. Liu , S. Zhang , K. Li , IEEE/ASME Trans. Mechatron. 2022, 27, 3908.

[163] X. Q. Tian , Q. Q. Quan , L. Wang , Q. Su , IEEE Access 2018, 6, 18975.

[164] Y. X. Liu , W. S. Chen , X. H. Yang , J. K. Liu , Ultrasonics 2015, 56, 551. 25454095 10.1016/j.ultras.2014.10.010

[165] Q. Su , Q. Quan , J. Deng , H. Yu , Sensors 2018, 18, 810. 29518964 10.3390/s18030810 PMC5876755

[166] Y. Wang , J. Deng , H. Li , X. Tian , W. Chen , Y. Liu , IEEE Trans. Ind. Electron. 2023, 70, 8235.

[167] D. Robles‐Cuenca , M. R. Ramirez‐Palma , V. Ruiz‐Diez , J. Hernando‐Garcia , J. L. Sanchez‐Rojas , Micromachines 2022, 13, 1815. 36363836 10.3390/mi13111815 PMC9692952

[168] D. Xu , Y. Liu , S. Shi , J. Liu , W. Chen , L. Wang , IEEE/ASME Trans. Mechatron. 2018, 23, 444.

[169] M. Takato , M. Tatani , H. Oku , Y. Okane , J. Tanida , S. Yamasaki , K. Saito , F. Uchikoba , Int. J. Adv. Rob. Syst. 2014, 11, 99.

[170] H. Fang , K. W. Wang , J. Sound Vib. 2017, 391, 153.

[171] X. Gao , Y. Liu , S. Zhang , J. Deng , J. Liu , IEEE/ASME Trans. Mechatron. 2022, 27, 3977.

[172] A. J. Fleming , Y. K. Yong , IEEE/ASME Trans. Mechatron. 2017, 22, 2611.

[173] Q. Chang , W. Chen , J. Liu , H. Yu , J. Deng , Y. Liu , Mech. Syst. Signal Process. 2021, 159, 107851.

[174] Q. Su , W. Chen , J. Deng , X. Tian , Y. Liu , Mech. Syst. Signal Process. 2021, 158, 107768.

[175] S. Zhang , H. Zhao , X. Ma , J. Deng , Y. Liu , IEEE Trans. Ind. Electron. 2023, 70, 8264.

[176] Y. Tian , K. Lu , F. Wang , C. Zhou , Y. Ma , X. Jing , C. Yang , D. Zhang , IEEE/ASME Trans. Mechatron. 2020, 25, 1322.

[177] C. Liang , F. Wang , Z. Huo , B. Shi , Y. Tian , X. Zhao , D. Zhang , IEEE Trans. Ind. Electron. 2020, 67, 6963.

[178] Z. Lyu , Q. Xu , IEEE Trans. Rob. 2023, 39, 470.

[179] D. Zhang , P. Li , J. Zhang , H. Chen , K. Guo , M. Ni , IEEE/ASME Trans. Mechatron. 2019, 24, 2097.

[180] X. Ma , Y. Liu , J. Liu , J. Deng , IEEE Trans. Rob. 2021, 38, 765.

[181] P. A. York , N. T. Jafferis , R. J. Wood , Smart Mater. Struct. 2018, 27, 015008.

[182] M. Leveziel , W. Haouas , G. J. Laurent , M. Gauthier , R. Dahmouche , Sci. Rob. 2022, 7, eabn4292. 10.1126/scirobotics.abn4292 36001685

[183] X. Ma , J. Liu , J. Deng , Q. Chang , Y. Liu , IEEE Trans. Ind. Electron. 2023, 70, 12660.

[184] X. Ma , Y. Liu , J. Deng , X. Gao , J. Cheng , Mech. Syst. Signal Process. 2023, 184, 109704.

[185] J. Deng , S. Zhang , Y. Li , X. Ma , X. Gao , H. Xie , Y. Liu , Smart Mater. Struct. 2022, 31, 045020.

[186] J. Kim , J.‐H. Lee , Smart Mater. Struct. 2005, 14, 934.

[187] O. Fuchiwaki , Precis. Eng. 2013, 37, 88.

[188] P. Q. Fan , H. N. Liu , L. L. Zheng , Smart Mater. Struct. 2021, 30, 035023.

[189] H. Yu , Y. Liu , J. Deng , S. Zhang , W. Chen , Sci. China: Technol. Sci. 2022, 66, 233.

[190] U. Simu , S. Johansson , J. Micromech. Microeng. 2002, 12, 582.

[191] S. D. de Rivaz , B. Goldberg , N. Doshi , K. Jayaram , J. Zhou , R. J. Wood , Sci. Rob. 2018, 3, eaau3038. 10.1126/scirobotics.aau3038 33141691

[192] T. Ho , S. Choi , S. Lee , in 2007 IEEE Int. Conf. on Robotics and Biomimetics (ROBIO), Vol. 1–5, IEEE, Piscataway, NJ 2007, p. 1160.

[193] A. A. Yumaryanto , J. B. An , L. L. Xin , Key Eng. Mater. 2006, 326‐328, 1435.

[194] S. Zhang , Y. Liu , J. Deng , X. Gao , J. Li , W. Wang , M. Xun , X. Ma , Q. Chang , J. Liu , W. Chen , J. Zhao , Nat. Commun. 2023, 14, 500. 36717566 10.1038/s41467-023-36243-3 PMC9887007

[195] L. Wang , H. Wang , Y. Zhang , Z. Qiu , T. Cheng , Rev. Sci. Instrum. 2023, 94, 025003. 36859050 10.1063/5.0134324

[196] S. M. Hua , Y. Q. Wang , X. J. Wang , G. M. Cheng , App. Mech. Mater. 2012, 101‐102, 164.

[197] I. Adibnazari , W. S. Nagel , K. K. Leang , Int. J. Intell. Rob. 2018, 2, 425.

[198] J. Li , J. Deng , S. Zhang , F. Che , Y. Liu , IEEE Trans. Ind. Electron. 2023. 10.1109/tie.2023.32705111.

[199] B. Zhong , B. Liu , Z. Jin , Z. Wang , L. Sun , Microsyst. Technol. 2019, 26, 437.

[200] X. Zhang , B. Zhong , B. Liu , Z. Jin , Z. Wang , L. Sun , Int. J. Precis. Eng. Manuf. 2021, 22, 473.

[201] J. Li , S. Zhang , Y. Liu , J. Deng , X. Ma , Smart Mater. Struct. 2022, 31, 095008.

[202] X. Gao , S. Zhang , J. Deng , Y. Liu , IEEE Trans. Ind. Electron. 2021, 68, 724.

[203] A. Kortschack , O. C. Hänßler , C. Rass , S. Fatikow , in Proc. of the 2003 IEEE/RSJ Int. Conf. on Intelligent Robots and Systems (IROS 2003), Vol. 1–4, IEEE, Piscataway 2003, p. 1895.

[204] J. Deng , S. Liu , Y. Liu , L. Wang , X. Gao , K. Li , IEEE Trans. Ind. Electron. 2022, 69, 3918.

[205] J. Deng , Y. Liu , J. Li , S. Zhang , K. Li , IEEE Trans. Ind. Electron. 2022, 69, 6429.

[206] H. Yu , Y. Liu , J. Deng , S. Zhang , W. Chen , Mech. Syst. Signal Process. 2022, 170, 108815.

[207] Z. Ding , J. Dong , X. Zhou , Z. Xu , W. Qiu , C. Shen , Mech. Syst. Signal Process. 2022, 181, 109494.

[208] X. Tian , W. Chen , B. Zhang , Y. Liu , IEEE Trans. Ind. Electron. 2022, 69, 10396.

[209] S. Zhang , Y. Liu , X. Gao , J. Deng , H. Yu , W. Chen , Int. J. Mech. Sci. 2022, 220, 107165.

[210] J. Deng , Y. X. Liu , J. Li , S. J. Zhang , H. Xie , IEEE/ASME Trans. Mechatron. 2022, 27, 4122.

[211] J. Deng , Y. Liu , S. Zhang , J. Li , IEEE/ASME Trans. Mechatron. 2021, 26, 2059.

[212] Q. Pan , Z. Huang , M. Zhao , L. Chen , Q. Huang , R. Li , Mech. Syst. Signal Process. 2023, 183, 109618.

[213] R. Bansevicius , A. Drukteiniene , G. Kulvietis , I. Tumasoniene , Int. J. Adv. Rob. Syst. 2013, 10, 219.

[214] H. Yu , Y. Liu , J. Deng , J. Li , S. Zhang , W. Chen , J. Zhao , Adv. Intell. Syst. 2022, 4, 2100142.

[215] B. Jia , L. Wang , R. Wang , J. Jin , Z. Zhao , D. Wu , Smart Mater. Struct. 2021, 30, 035016.

[216] H. Li , J. Deng , Y. Liu , Ultrasonics 2023, 131, 106957. 36812818 10.1016/j.ultras.2023.106957

[217] T. Cheng , M. He , H. Li , X. Lu , H. Zhao , H. Gao , IEEE Trans. Ind. Electron. 2017, 64, 5545.

[218] Z. Li , Z. Wang , H. Han , H. Sun , Rev. Sci. Instrum. 2021, 92, 025004. 33648056 10.1063/5.0030599

[219] V. Ruiz‐Díez , J. L. García‐Caraballo , J. Hernando‐García , J. L. Sánchez‐Rojas , Actuators 2021, 10, 335.

[220] X. Zeng , Y. Wu , S. Han , Y. Liu , H. Xiu , F. Tian , L. Ren , Micromachines 2021, 12, 1577. 34945427 10.3390/mi12121577 PMC8704217

[221] J. Qu , C. B. Teeple , B. Zhang , K. R. Oldham , in 2018 Int. Conf. on Manipulation, Automation and Robotics at Small Scales (MARSS) (Eds: S. Haliyo , A. Sill , F. Arai , S. Fatikow ), IEEE, Piscataway, NJ 2018.

[222] A. A. Calderón , Y. Chen , X. Yang , L. Chang , X.‐T. Nguyen , E. Singer , N. O. Pérez‐Arancibia , in 2019 19th Int. Conf. on Advanced Robotics (ICAR), IEEE, Piscataway, NJ 2019, p. 747.

[223] C. Wang , H. Li , Z. Zhang , P. Yu , L. Yang , J. Du , Y. Niu , J. Jiang , J. Intell. Rob. Syst. 2022, 105, 56.

[224] Y. Liu , Y. Chen , B. Feng , D. Wang , T. Liu , H. Zhou , H. Li , S. Qu , W. Yang , IEEE Rob. Autom. Lett. 2022, 7, 6758.

[225] Z. Huang , G. Shao , L. Li , Prog. Mater. Sci. 2023, 131, 101020.

[226] J. Fan , L. Zhang , S. Wei , Z. Zhang , S.‐K. Choi , B. Song , Y. Shi , Mater. Today 2021, 50, 303.

[227] A. Reiser , L. Koch , K. A. Dunn , T. Matsuura , F. Iwata , O. Fogel , Z. Kotler , N. Zhou , K. Charipar , A. Pique , P. Rohner , D. Poulikakos , S. Lee , S. K. Seol , I. Utke , C. van Nisselroy , T. Zambelli , J. M. Wheeler , R. Spolenak , Adv. Funct. Mater. 2020, 30, 1910491. 32684902 10.1002/adfm.201910491 PMC7357576

[228] J. Qu , B. Zhang , K. R. Oldham , Int. J. Intell. Rob. 2018, 2, 400.

[229] K. Patel , J. Qu , K. R. Oldham , in 2018 Int. Conf. on Manipulation, Automation and Robotics at Small Scales (MARSS) (Eds: S. Haliyo , A. Sill , F. Arai , S. Fatikow ), IEEE, Piscataway, NJ 2018.

[230] B. Zhang , J. Qu , K. R. Oldham , in 2018 IEEE/ASME Int. Conf. on Advanced Intelligent Mechatronics (AIM), IEEE, Piscataway, NJ 2018, p. 718.

[231] A. G. Dharmawan , H. H. Hariri , G. S. Soh , S. Foong , K. L. Wood , J. Mech. Rob. 2018, 10, 021003.

[232] J. B. Gafford , S. B. Kesner , R. J. Wood , C. J. Walsh , in Proc. of the ASME Int. Design Engineering Technical Conf. and Computers and Information in Engineering Conf., Vol. 6A, ASME, New York 2013, V06AT07A011.

[233] J. B. Gafford , S. B. Kesner , R. J. Wood , C. J. Walsh , in 2013 IEEE/RSJ Int. Conf. on Intelligent Robots and Systems (IROS) (Ed: N. Amato ), IEEE, Piscataway, NJ 2013, p. 2552.

[234] A. T. Baisch , O. Ozcan , B. Goldberg , D. Ithier , R. J. Wood , Int. J. Rob. Res. 2014, 33, 1063.

[235] S. M. Felton , K. P. Becker , D. M. Aukes , R. J. Wood , J. Micromech. Microeng. 2015, 25, 085004.

[236] C. D. Onal , R. J. Wood , D. Rus , in 2011 IEEE Int. Conf. on Robotics and Automation (ICRA), IEEE, Piscataway, NJ 2011, p. 4608.

[237] P. S. Gollnick , S. P. Magleby , L. L. Howell , J. Micromech. Microeng. 2011, 133, S259.

[238] J. O. Jacobsen , B. G. Winder , L. L. Howell , S. P. Magleby , J. Mech. Rob. 2010, 2, 011003.

[239] D. M. Aukes , B. Goldberg , M. R. Cutkosky , R. J. Wood , Smart Mater. Struct. 2014, 23, 094013.

[240] T. George , A. K. Dutta , M. S. Islam , D. M. Aukes , R. J. Wood , Proc. SPIE 2015, 9467, 94671B.

[241] N. T. Jafferis , M. J. Smith , R. J. Wood , Smart Mater. Struct. 2015, 24, 065023.

[242] P. S. Sreetharan , J. P. Whitney , M. D. Strauss , R. J. Wood , J. Micromech. Microeng. 2012, 22, 055027.

[243] K. Jayaram , J. Shum , S. Castellanos , E. F. Helbling , R. J. Wood , in 2020 IEEE Int. Conf. on Robotics and Automation (ICRA), IEEE, Piscataway, NJ 2020, p. 10305.

[244] K. L. Hoffman , R. J. Wood , Auton. Rob. 2011, 31, 103.

[245] K. L. Hoffman , R. J. Wood , in 2012 4th IEEE RAS & EMBS Int. Conf. on Biomedical Robotics and Biomechatronics (BioRob) (Eds: J. Desai , L. Jay , L. Zollo ), IEEE, Piscataway, NJ 2012, p. 1052.

[246] K. T. Nguyen , S. Y. Ko , J.‐O. Park , S. Park , IEEE/ASME Trans. Mechatron. 2015, 20, 2962.

[247] H. Lim , S.‐W. Kim , J.‐B. Song , Y. Cha , IEEE Access 2021, 9, 145477.

[248] H. Jalili , H. Salarieh , G. Vossoughi , J. Sound Vib. 2018, 436, 81.

[249] C. Shen , S. Yu , J. Luo , K. R. Oldham , Appl. Sci. 2021, 11, 6276.

[250] W. Zhao , A. Ming , M. Shimojo , Appl. Bionics Biomech. 2018, 2018, 5697408. 30598697 10.1155/2018/5697408 PMC6288587

[251] T. E. Hooper , J. I. Roscow , A. Mathieson , H. Khanbareh , A. J. Goetzee‐Barral , A. J. Bell , J. Eur. Ceram. Soc. 2021, 41, 6115.

[252] X. Xin , Y. Yu , J. Wu , X. Gao , Z. Li , X. Yi , W. Chen , S. Dong , Sens. Actuators, A 2020, 309, 112036.

[253] Z. Li , X. Gao , J. Yang , X. Xin , X. Yi , L. Bian , S. Dong , Adv. Sci. 2020, 7, 2001155. 10.1002/advs.202001155 PMC7435238 32832366

[254] G. L. Messing , S. Poterala , Y. Chang , T. Frueh , E. R. Kupp , B. H. Watson , R. L. Walton , M. J. Brova , A.‐K. Hofer , R. Bermejo , R. J. Meyer , J. Mater. Res. 2017, 32, 3219.

[255] X. Song , Z. Chen , L. Lei , K. Shung , Q. Zhou , Y. Chen , Rapid Prototyping J. 2017, 23, 44.

[256] K. Zheng , D. Ding , Y. Quan , J. Zhuang , C. Fei , J. Zhao , L. Wang , T. Zhao , Z. Wang , M. Liu , Z. Jiang , Z. Jiang , L. Wen , S. Wu , W. Ren , J. Eur. Ceram. Soc. 2023, 43, 2408.

[257] W. Chen , F. Wang , K. Yan , Y. Zhang , D. Wu , Ceram. Int. 2019, 45, 4880.

[258] C. Chen , X. Wang , Y. Wang , D. Yang , F. Yao , W. Zhang , B. Wang , G. A. Sewvandi , D. Yang , D. Hu , Adv. Funct. Mater. 2020, 30, 2005141.

[259] J. Cheng , Y. Chen , J. W. Wu , X. R. Ji , S. H. Wu , Sensors 2019, 19, 4078.31547206

[260] J. Liu , X. Gao , H. Jin , K. Ren , J. Guo , L. Qiao , C. Qiu , W. Chen , Y. He , S. Dong , Z. Xu , F. Li , Nat. Commun. 2022, 13, 6567. 36323672 10.1038/s41467-022-34231-7 PMC9630418

[261] J. Yang , Z. Li , X. Xin , X. Gao , X. Yuan , Z. Wang , Z. Yu , X. Wang , J. Zhou , S. Dong , Sci. Adv. 2019, 5, eaax1782. 31976367 10.1126/sciadv.aax1782 PMC6957242

[262] R. Hensleigh , H. Cui , Z. Xu , J. Massman , D. Yao , J. Berrigan , X. Zheng , Nat. Electron. 2020, 3, 216.

[263] H. Cui , D. Yao , R. Hensleigh , H. Lu , A. Calderon , Z. Xu , S. Davaria , Z. Wang , P. Mercier , P. Tarazaga , X. R. Zheng , Science 2022, 376, 1287. 35709267 10.1126/science.abn0090

[264] Y.‐Z. Ji , Z. Wang , B. Wang , Y. Chen , T. Zhang , L.‐Q. Chen , X. Song , L. Chen , Adv. Eng. Mater. 2017, 19, 1600803.

[265] Z. Zheng , B. Wang , H. Yin , Y. Chen , Y. Bao , Y. Guo , Adv. Funct. Mater. 2023, 2302648. 10.1002/adfm.202302648.

[266] L. Zhang , S. Li , Z. Zhu , G. Rui , B. Du , D. Chen , Y. F. Huang , L. Zhu , Adv. Funct. Mater. 2023, 2301302. 10.1002/adfm.202301302.

[267] J. Liang , Y. Wu , Z. Shao , J. K. Yim , R. Xu , Y. Song , M. Qi , J. Zhong , M. Zhang , X. Wang , in 2019 IEEE 32nd Int. Conf. on Micro Electro Mechanical Systems (MEMS), IEEE, Piscataway, NJ 2019, p. 1041.

[268] S. Scheffler , P. Poulin , ACS Appl. Mater. Interfaces 2022, 14, 16961. 35404561 10.1021/acsami.1c24611

[269] J. Liang , Y. Wu , J. K. Yim , H. Chen , Z. Miao , H. Liu , Y. Liu , Y. Liu , D. Wang , W. Qiu , Z. Shao , M. Zhang , X. Wang , J. Zhong , L. Lin , Sci. Rob. 2021, 6, eabe7906. 10.1126/scirobotics.abe7906 34193563

[270] Y. Wu , K. Y. Ho , K. Kariya , R. Xu , W. Cai , J. Zhong , Y. Ma , M. Zhang , X. Wang , L. Lin , in 2018 IEEE Micro Electro Mechanical Systems (MEMS), IEEE, Piscataway, NJ 2018, p. 581.

[271] T. Park , Y. Cha , Sci. Rep. 2019, 9, 14700. 31605017 10.1038/s41598-019-51308-4 PMC6788992

[272] Y. Wu , J. K. Yim , J. Liang , Z. Shao , M. Qi , J. Zhong , Z. Luo , X. Yan , M. Zhang , X. Wang , R. S. Fearing , R. J. Full , L. Lin , Sci. Rob. 2019, 4, eaax1594. 10.1126/scirobotics.aax1594 33137774

[273] D. Hu , J. Lou , T. Chen , Y. Yang , C. Xu , H. Chen , Y. Cui , Mech. Syst. Signal Process. 2021, 153, 107538.

[274] A. S. Barbosa , L. Z. Tahara , M. M. da Silva , J. Vib. Control 2021, 29, 411.

[275] A. S. Barbosa , M. M. Da Silva , in 2021 IEEE Int. Conf. on Robotics and Biomimetics (ROBIO), IEEE, Piscataway, NJ 2021, p. 118.

[276] A. Erturk , G. Delporte , Smart Mater. Struct. 2011, 20, 125013.

[277] Y. Nagata , S. Park , A. Ming , M. Shimojo , in 2008 IEEE/ASME Int. Conf. on Advanced Intelligent Mechatronics, IEEE, Piscataway, NJ 2008, p. 955.

[278] F. A. Naser , H. A. Jaber , M. T. Rashid , B. H. Jasim , IFAC‐PapersOnLine 2021, 54, 117.

[279] W. Zhao , T. Osaka , A. Ming , M. Shimojo , in 2011 IEEE Int. Conf. on Robotics and Biomimetics (IEEE‐ROBIO), IEEE, Piscataway, NJ 2011, p. 118.

[280] H. Meng , J. Lou , T. Chen , C. Xu , H. Chen , Y. Yang , Y. Cui , Smart Mater. Struct. 2021, 30, 035001.

[281] H. Cheng , Z. Zheng , P. Kumar , Y. Chen , M. Chen , in 2022 IEEE Applied Power Electronics Conf. and Exposition (APEC), IEEE, Piscataway, NJ 2022, p. 1338.

[282] Z. Zheng , P. Kumar , Y. Chen , H. Cheng , S. Wagner , M. Chen , N. Verma , J. Sturm , in 2022 IEEE Int. Conf. on Robotics and Automation (ICRA 2022), IEEE, Piscataway, NJ 2022, p. 5199.

[283] R. Bansevicius , V. Blechertas , J. Electroceram. 2007, 20, 221.

[284] S. Fatikow , V. Eichhorn , C. Stolle , T. Sievers , M. Jähnisch , Mechatronics 2008, 18, 370.

[285] C. Stolle , S. Fatikow , in 2007 IEEE 22nd Int. Symp. on Intelligent Control, IEEE, Piscataway, NJ 2007, p. 664.

[286] R. St. Pierre , S. Bergbreiter , Annu. Rev. Control Rob. Auton. Syst. 2019, 2, 231.

[287] N. T. Jafferis , M. Lok , N. Winey , G.‐Y. Wei , R. J. Wood , Smart Mater. Struct. 2016, 25, 055033.

[288] K. Teichert , K. Oldham , in Proceedings of the ASME 11th Annual Dynamic Systems and Control Conf., Vol. 2, ASME, New York 2018, p. V002T19A004.

[289] R. Zhu , Y. Zhang , H. Wang , Micromachines 2022, 13, 1184. 36014106 10.3390/mi13081184 PMC9413211

[290] M. Rubenstein , C. Ahler , N. Hoff , A. Cabrera , R. Nagpal , Rob. Auton. Syst. 2014, 62, 966.

[291] X. Huang , Y. Hu , J. Ma , J. Li , H. Lin , J. Wen , Smart Mater. Struct. 2021, 30, 095014.

[292] B. Zhong , J. Zhu , Z. Jin , H. He , L. Sun , Z. Wang , Precis. Eng. 2019, 55, 260.

[293] M. Lok , E. F. Helbling , X. Zhang , R. Wood , D. Brooks , G.‐Y. Wei , IEEE Trans. Power Electron. 2018, 33, 3180.

[294] M. Karpelson , G.‐Y. Wei , R. J. Wood , Sens. Actuators, A 2012, 176, 78.

[295] C. Yang , K. Youcef‐Toumi , Mech. Syst. Signal Process. 2022, 171, 108885.

[296] W. Mu , M. Li , E. Chen , Y. Yang , J. Yin , X. Tao , G. Liu , R. Yin , Adv. Funct. Mater. 2023, 33, 2300516.

[297] W. Wang , J. Deng , Y. Liu , S. Zhang , J. Li , X. Gao , Smart Mater. Struct. 2022, 31, 075004.

[298] B. J. Nelson , S. M. Martel , J.‐M. Breguet , W. Garcia de Quevedo , I. W. Hunter , Proc. SPIE 2000, 4194, 168.

[299] R. Brühwiler , B. Goldberg , N. Doshi , O. Ozcan , N. Jafferis , M. Karpelson , R. J. Wood , in 2015 IEEE/RSJ Int. Conf. on Intelligent Robots and Systems (IROS), IEEE, Piscataway, NJ 2015, p. 5727.

[300] M. Karpelson , B. H. Waters , B. Goldberg , B. Mahoney , O. Ozcan , A. Baisch , P.‐M. Meyitang , J. R. Smith , R. J. Wood , in 2014 IEEE Int. Conf. on Robotics and Automation (ICRA), IEEE, Piscataway, NJ 2014, p. 2384.

[301] B. Goldberg , R. Zufferey , N. Doshi , E. F. Helbling , G. Whittredge , M. Kovac , R. J. Wood , IEEE Rob. Autom. Lett. 2018, 3, 987.

[302] B. Goldberg , N. Doshi , R. J. Wood , in 2017 IEEE Int. Conf. on Robotics and Automation (ICRA), IEEE, Piscataway, NJ 2017, p. 3538.

[303] B. Goldberg , N. Doshi , K. Jayaram , J.‐S. Koh , R. J. Wood , in 2017 IEEE/RSJ Int. Conf. on Intelligent Robots and Systems (IROS) (Eds: A. Bicchi , A. Okamura ), IEEE, Piscataway, NJ 2017, p. 3964.

[304] W. Osten , R. Estana , M. Kujawinska , H. Woern , K. Creath , Proc. SPIE 2003, 5144, 431.

[305] J. Otero , A. Saiz , J. Brufau , J. Colomer , R. Ruiz , J. Lopez , P. Miribel , M. Puig , J. Samitier , in 2006 1st IEEE/RAS‐EMBS Int. Conf. on Biomedical Robotics and Biomechatronics, Vol. 1–3, IEEE, Piscataway, NJ 2006, p. 1078.

[306] E. Chen , Y. Yang , M. Li , B. Li , G. Liu , W. Mu , R. Yin , Adv. Sci. 2023, 10, 2300673. 10.1002/advs.202300673 PMC10369280 37163730

[307] Z. Li , X. Yi , R. Zhu , Z. Yu , X. Yuan , M. PourhosseiniAsl , S. Dong , Research 2023, 6, 0156. 37287892 10.34133/research.0156 PMC10243895

[308] S. Duque Tisnes , A. Tasneem , L. Petit , C. Prelle , Appl. Sci. 2021, 11, 11980.

[309] S. Zhou , W. Zhang , Y. Zou , B. Ou , Z. Xun , Microsyst. Technol. 2017, 24, 943.

[310] Z. Zheng , Y. Zhao , G. Wang , J. Bionic Eng. 2023, 20, 1481.

[311] J. Lu , Z. Miao , Z. Wang , Y. Liu , D. Zhu , J. Yin , F. Tang , X. Wang , W. Ding , M. Zhang , Nano Res. 2022, 16, 4970.

[312] Q. Lu , Z. Sun , H. Yan , J. Zhang , J. Zhang , J. Yang , J. Intell. Mater. Syst. Struct. 2022, 34, 1276.

[313] T. Abondance , K. Jayaram , N. T. Jafferis , J. Shum , R. J. Wood , IEEE Rob. Autom. Lett. 2020, 5, 4407.

[314] J. Xing , C. Ning , Y. Liu , I. Howard , Front. Mech. Eng. 2022, 17, 41.

[315] A. Aabid , M. Hrairi , S. J. Mohamed Ali , Y. E. Ibrahim , ACS Omega 2023, 8, 2844. 36713708 10.1021/acsomega.2c06573 PMC9878659

[316] A. Eisinberg , I. Karjalainen , A. Menciassi , P. Dario , J. Seyfried , R. Estana , H. Woern , Assem. Autom. 2007, 27, 123.

[317] C. B. Qin , L. F. Tian , Q. L. Du , Q. Zhang , in Proc. of Advanced Materials Research, (Eds: X. Huang , X. Zhu , K. Xu , J. Wu ), Scientific.Net, Taiyuan, PEOPLES R CHINA 2014, Vol 1049–1050, p. 1116.

[318] A. Kortschack , S. J. J. o. M. Fatikow , J. Micromechatronics 2004, 2, 249.

[319] C.‐H. Yun , L. Y. Yeo , J. R. Friend , B. Yan , Appl. Phys. Lett. 2012, 100, 164101.

[320] D. M. Ohmi Fuchiwaki , H. Aoyama , in 7th Int. Conf. on Mechatronics Technology, IEEE, Piscataway, NJ 2003, p. 121.

[321] B. Komati , C. Clevy , P. Lutz , IEEE/ASME Trans. Mechatron. 2016, 21, 2039.

[322] S. Fatikow , V. Eichhorn , Proc. Inst. Mech. Eng., Part C 2008, 222, 1353.

[323] L. Dong , S. Fatikow , Y. Katagiri , V. Eichhorn , A. Sill , E. Higurashi , H. Toshiyoshi , A. Steinecker , C. Meyer , Y.‐A. Peter , L. Occhupinti , S. Fahlbusch , I. Utke , P. Bøggild , J. M. Breguet , R. Kaufmann , M. Zadrazil , W. Barth , Proc. SPIE 2007, 6717, 67170J.

[324] V. Eichhorn , S. Fatikow , T. Wortmann , C. Stolle , C. Edeler , D. Jasper , O. Sardan , P. Boggild , G. Boetsch , C. Canales , in 2009 IEEE Int. Conf. on Robotics and Automation (ICRA), Vol. 1–7, IEEE, Piscataway, NJ 2009, p. 1643.

[325] O. Fuchiwaki , Y. Tanaka , H. Notsu , T. Hyakutake , Microfluid. Nanofluid. 2018, 22, 80.

[326] Z. Li , X. Yi , R. Zhu , Z. Yu , X. Yuan , M. PourhosseiniAsl , S. Dong , Research 2023, 6, 0156. 37287892 10.34133/research.0156 PMC10243895

[327] Y. Hu , J. Ma , Y. Zhang , J. Li , Y. Hu , J. Wen , Mech. Syst. Signal Process. 2021, 157, 107743.

[328] B. J. Nelson , F. Schmoeckel , J.‐M. Breguet , S. Fahlbusch , J. Seyfried , A. Buerkle , S. Fatikow , Proc. SPIE 2000, 4194, 129.

[329] Y. Wei , Q. Xu , IEEE Sens. J. 2019, 19, 6012.

[330] M. Xie , S. Yu , H. Lin , J. Ma , H. Wu , IEEE Trans. Circuits Syst. I: Regul. Pap. 2020, 67, 3199.

[331] W. Meinhold , D. E. Martinez , J. Oshinski , A.‐P. Hu , J. Ueda , IEEE Trans. Biomed. Eng. 2021, 68, 807. 32870782 10.1109/TBME.2020.3020926

[332] W. Johnson , C. Dai , J. Liu , X. Wang , D. K. Luu , Z. Zhang , C. Ru , C. Zhou , M. Tan , H. Pu , S. Xie , Y. Peng , J. Luo , Y. Sun , IEEE Trans. Biomed. Eng. 2018, 65, 678. 28600237 10.1109/TBME.2017.2713302

[333] T. Zhang , L. Gong , S. Wang , S. Zuo , Ann. Biomed. Eng. 2019, 48, 413. 31531791 10.1007/s10439-019-02358-2

[334] O. Fuchiwaki , D. Misaki , C. Kanamori , H. Aoyama , J. Micro‐Nano Mechatron. 2008, 4, 85.

[335] A. Kortschack , A. Shirinov , T. Trüper , S. Fatikow , Tec. Autom. 2005, 23, 419.

[336] T. Truper , A. Kortschack , M. Jahnisch , H. Hulsen , S. Fatikow , IEE Proc. Nanobiotechnol. 2004, 151, 145. 16475859 10.1049/ip-nbt:20040839

[337] R. Casanova , A. Saiz‐Vela , A. Arbat , J. Colomer , P. Miribel , A. Dieguez , M. Puig , J. Samitier , in 1st IEEE/RAS‐EMBS Int. Conf. on Biomedical Robotics and Biomechatronics, IEEE, Piscataway, NJ 2006, p. 13.

[338] F. Tagliareni , M. Nierlich , O. Steinmetz , T. Velten , J. Brufau , J. Lopez‐Sanchez , M. Puig‐Vidal , J. Samitier , in 2005 IEEE/RSJ Int. Conf. on Intelligent Robots and Systems, Vol. 1–4, IEEE, Piscataway, NJ 2005, p. 426.

[339] J. Brufau , M. Puig‐Vidal , J. Lopez‐Sanchez , J. Samitier , N. Snis , U. Simu , S. Johansson , W. Driesen , J.‐M. Breguet , J. Gao , in 2005 IEEE Int. Conf. on Robotics and Automation (ICRA), IEEE, Vol. 1–4, 2005, p. 844.

[340] O. Fuchiwaki , A. Ito , D. Misaki , H. Aoyama , in 2008 IEEE Int. Conf. on Robotics and Automation (ICRA), Vol. 1–9, IEEE, Piscataway, NJ 2008, p. 893.

[341] H. Wörn , F. Schmoeckel , A. Buerkle , J. Samitier , M. Puig‐Vidal , S. A. Johansson , U. Simu , J.‐U. Meyer , M. Biehl , Proc. SPIE 2001, 4568, 175.

[342] H. Aoyama , O. Fuchiwaki , D. Misaki , T. Usuda , in 2006 IEEE Conf. on Robotics, Automation and Mechatronics (ICRA), Vol. 1–2, IEEE, Piscataway, NJ 2006, , p. 75.

